# Back-Contact Perovskite Solar Cell Modules Fabricated via Roll-to-Roll Slot-Die Coating: Scale-Up toward Manufacturing

**DOI:** 10.1021/acsaem.4c02734

**Published:** 2025-02-18

**Authors:** Dominic Blackburn, Nathan S. Hill, Christopher J. Wood, Tamilselvan Velusamy, Balder A. Nieto-Díaz, Caitlin Woolley, Andy Brown, Loukas Zampelis, Trevor McArdle, Molly Worth, Timothy Thornber, Ibrahim Albariqi, Rachel C. Kilbride, Tingxiang Yang, C. Neil Hunter, Graham J. Leggett, George Koutsourakis, James C. Blakesley, Fernando A. Castro, David Beynon, Trystan M. Watson, Dumitru Sirbu, David G. Lidzey

**Affiliations:** † Department of Physics and Astronomy, Hicks Building, Hounsfield Road, Sheffield S3 7RH, United Kingdom; ‡ Power Roll Ltd, Jade Business Park, Spring Road, Seaham SR7 9DR, United Kingdom; § SPECIFIC, Swansea University, Bay Campus, Fabian Way, Swansea SA1 8EN, United Kingdom; ∥ Physics Department, Faculty of Science, Al-Baha University, Alaqiq 65779-7738, Kingdom of Saudi Arabia; ⊥ Department of Chemistry, Dainton Building, University of Sheffield, Brook Hill, Sheffield S3 7HF, United Kingdom; # School of Biosciences, University of Sheffield, Sheffield S10 2TN, United Kingdom; ∇ National Physical Laboratory, Hampton Road, Teddington TW11 0LW, United Kingdom

**Keywords:** Perovskite, solar cell, back-contact, scaled, roll-to-roll, commercialization

## Abstract

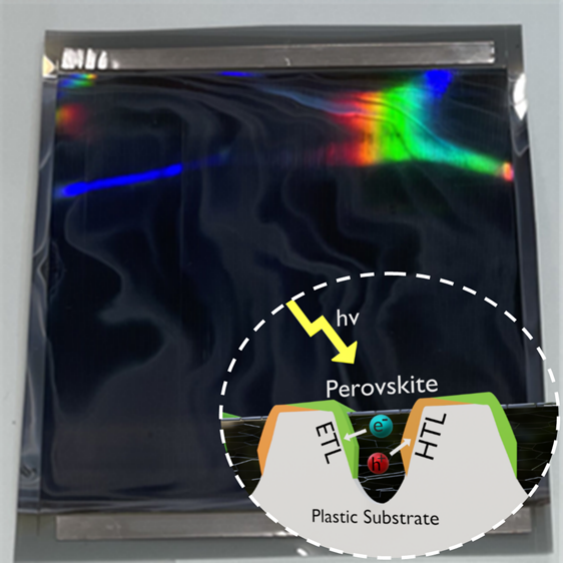

We fabricate a type of back-contact perovskite solar cell based on 1.5 μm-width grooves that are embossed into a plastic film whose opposing “walls” are selectively coated with either n- or p-type contacts. A perovskite precursor solution is then deposited into the grooves, creating individual photovoltaic devices. Each groove device is series-connected to its neighbors, creating minimodules consisting of hundreds of connected grooves. Here, we report on the fabrication of groove-based devices using slot-die coating to deposit the perovskite precursor and explore the structure of the perovskite in the grooves using a range of microscopy and spectroscopy techniques. Significantly, our devices do not contain any expensive or scarce elements such as indium, indicating that this technology is both sustainable and low-cost. Furthermore, all coating processes explored here were performed using roll-to-roll processing techniques. Our technology is therefore completely scalable and is consistent with high-throughput, low-cost manufacturing.

## Introduction

Solar cells based on metal-halide perovskites have undergone rapid development in recent years, with their power conversion efficiencies (PCE) increasing from 3.8% to over 26%.^1^ Such high efficiencies are the result of low exciton binding energies, long charge-carrier diffusion lengths and band gaps that are tunable to near IR wavelengths.^2−4^ As perovskites have intrinsically low materials processing costs, perovskite solar cells (PSCs) now appear to be a potential future competitor to devices based on silicon.^4,5^

Almost all PSC devices reported are based on a planar layer-by-layer architecture that is deposited on a transparent conductive oxide (TCO) coated substrate. While such devices can have very high efficiencies, they also have limitations. For example, parasitic optical absorption within the charge transporting and contact layers can reduce the maximum attainable short-circuit current (*J*_sc_). Each layer in a multilayer stack must also be deposited from a solvent that is orthogonal to the preceding layers to avoid their resolubilization; this can limit the range of solvents available to process devices and reduce the range of processing conditions that can be used. Planar architecture PSCs typically also include TCOs such as Indium Tin Oxide (ITO) as their light-facing electrode. Although ITO combines high conductivity and transparency, supplies of ITO are at increasing risk due to the growing scarcity of indium and current world geopolitics.^6,7^ We note that indium is primarily extracted as a byproduct of zinc mining and is currently valued at around $670/kg, compared to $16/kg for nickel, another commonly used conductor. If the demand for indium exceeds that of zinc, it will be necessary to directly mine indium; a process that is less economically viable and is likely to further inflate materials costs.^8,9^ Indeed, if PSC production were to increase to a point where the global photovoltaic fleet provided terawatts of power per year, the annual production of indium would need to increase by 200%.^10^ Though alternatives to ITO exist and have been used in solar-cell devices (such as fluorine-doped tin oxide [FTO]), these materials often require high deposition temperatures, making them incompatible with deposition onto flexible plastic substrates. High process temperatures also increase both production time and cost.^11^ For such reasons, there is significant interest in the development of solar cell technologies that do not rely on a steady supply of indium.

Back-contact solar cells represent an alternative to traditional planar architecture devices. In general, back-contact devices have all electrode and transport layers positioned at the rear of the device, with the active layer at the front of the device thus directly absorbing incident light. This type of structure has the advantage of potentially eliminating parasitic optical absorbance from device contacts and charge transport layers. Back-contact architectures were initially developed for silicon solar cells in the 1970s and were first used in perovskite solar cells in 2016, with a PCE of 6.5% reported.^12,13^ Here, devices were composed of “fingers” of a hole transport layer that were electrically isolated from a planar electron transport layer, with a perovskite coating the whole structure. In the following years, several back-contact PSC architectures have been demonstrated, including interdigitated, quasi-interdigitated, honeycomb and groove.^13−16^

A key issue in the development of back-contact PSCs is the necessity to physically separate electron and hole transport layers by a distance that is commensurate with charge-carrier diffusion lengths, typically a few hundred nanometers in perovskites.^17−20^ If charge contacts are separated by a distance greater than this, device efficiency is reduced due to charge-carrier recombination losses.^21^ Thus, back-contact perovskite devices will require the use of submicron scale lithography techniques to define charge contacts, with this process being scalable for high-volume manufacture. Several lithography techniques have so far been explored; for example, the use of a self-assembly technique has been used to produce a honeycomb structured back-contact PSC in which the distance between each pore was approximately 7 μm. This design allowed devices to be created having champion PCEs of 11.2%.^22^ Further progress was made by the same group using cracked film lithography, where a lift-off technique was used to fill the cracks in a tin oxide film with nickel oxide and an insulator. When used in a back-contact configuration, PSCs were realized with reverse sweep efficiencies of over 6%.^23^ In the same year, Deng et al. used lithography with polystyrene microspheres adsorbed onto a tin oxide film to template a honeycomb structure that could then be used as an evaporation mask for an insulating layer and a top electrode. This method produced back-contact devices with a PCE of 8.9%.^24^ Of the various commonly used deposition techniques used to create back-contact devices, only one has been reported that involves the use of a truly scalable process (slot-die coating) with devices fabricated having PCEs of 0.18%.^25^

In 2019, we reported a novel back-contact architecture based on a V-shaped microgroove structure.^16^ In brief, 1.6 μm wide grooves were embossed into a plastic film and had electrodes and transport layers coated onto their opposing walls, with a perovskite then coated over the film surface. Using this structure, single groove efficiencies were demonstrated with a PCE of 7.03%, and serially connected micromodules comprising 16 grooves achieved a PCE of 2.63%.

In this work, we make a significant advance on our previous work and demonstrate a significant scale-up of our technology, creating micromodules composed of up to 362 serially connected grooves. Such devices also have significantly enhanced performance, realizing stabilized PCEs of up to 12.8%. This enhancement in performance has resulted from a detailed device optimization program in which a large parameter space relating to both the preparation of the patterned substrate and the techniques used to deposit the perovskite has been explored.

Here, we discuss the use of a range of techniques to explore the structure and operation of our devices to verify the structure of the electrodes and charge-transport layers deposited onto the opposing groove walls, including Scanning Electron Microscopy (SEM) and nanofocus X-ray fluorescence (XRF) mapping. This latter technique, together with Atomic Force Microscopy (AFM) allows us to characterize the crystallinity, topography, and size of perovskite grains within the grooves. We also explore photocurrent generation via photocurrent mapping and fluorescence decay lifetime measurements and use this to understand the functionality of the devices. Critically, we also show that the flexible, back-contact perovskite solar module devices we create can be fabricated using fully scalable, roll-to-roll deposition processes. Significantly, all techniques used in the processing of groove-based modules, including embossing, are fully compatible with upscaled manufacture, with the titanium and nickel metal contacts used here being significantly cheaper than Indium.

## Results and Discussion

### Groove Structure and Fabrication

**Figure 1:**
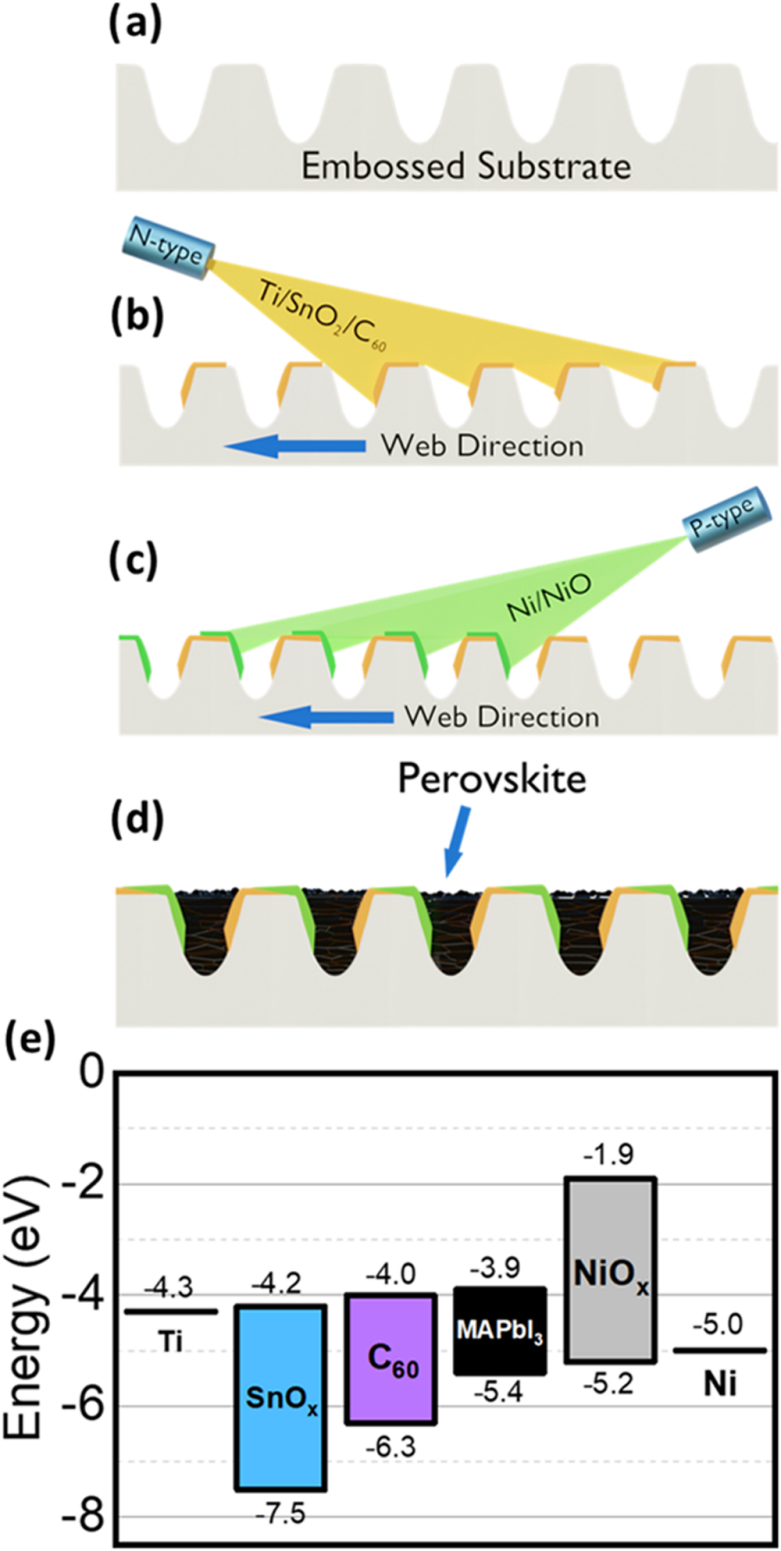
Schematic of the manufacture of a groove cascade showing (a) groove embossed into PET: acrylic substrate; (b, c) directional evaporation demonstrating the “self-shading” technique; (d) cross-section of complete groove after perovskite deposition; (e) Band diagram built from UPS, XPS and UV–visible spectrum data in S3, S4 and S5.

The structure of the devices explored here is schematically illustrated in Figure 1d. Briefly, fabrication begins with a PET: acrylic roll onto which a series of 1.5 μm-width grooves are embossed using a UV-curable acrylic. As we describe below, the back-contact architecture developed is based on coating opposing groove walls with electron- or hole-selective contacts. Here, the effective collection of charges without significant recombination effects requires the physical separation of the opposing contacts to be commensurate with typical carrier diffusion lengths. In the perovskite used in our devices (MAPbI_3_), a range of carrier diffusion lengths have been reported, ranging from 0.1 to 2 μm, with this length being dependent on process conditions.^26−28^ In an ideal structure, the widths of the grooves would match carrier diffusion lengths, however, creating submicron features over large areas is challenging. We have therefore adopted a groove width of around 1.5 μm; empirically we find this both results in efficient charge-carrier extraction and allows grooves to be reliably embossed over large areas having a consistent width and feature size.

The embossing process was undertaken at high volume under a commercial contract. In contrast to our previous work in which we used substrates embossed with a V-shaped groove, here we use grooves that have a U-shaped cross-section.^16^ This change in groove structure was based on the finding that U-shaped grooves are generally easier to emboss with high fidelity. This embossed roll was then loaded into a vacuum deposition chamber on a spool and was passed at a web speed of 5–15 mm/s in front of a series of deposition sources. Here, the coating selectivity of either wall was achieved using a directional deposition technique as shown in Figure 1b,c. To do this, an evaporation source was oriented at an oblique angle to the surface. This resulted in a “self-shading” effect that allowed each side of the groove walls to be coated by a contact material without coating the opposing wall, as previously reported.^16^ When selecting contact materials, it was necessary to identify materials having suitable electronic properties that could be deposited by either electron-beam (e-beam) or thermal evaporation. Attention was also paid to materials that were known to form stable contacts with perovskites, had low cost (to be compatible with a high-throughput manufacturing process), and did not require any high-temperature processes to be compatible with plastic substrates. For these reasons, we avoided commonly used contact materials such as gold, silver and ITO due to their cost, stability and processing requirements, respectively.

The n-type (electron extracting) contact deposited was composed of a Ti/SnO_2_/C_60_ multilayer, with the p-type (hole extracting) contact composed of Ni/NiO. All metals and metal oxides were deposited using e-beam evaporation with the SnO_2_ and NiO deposited by a reactive deposition in which a low partial pressure of oxygen was maintained in the chamber to oxidize the deposited material.^29,30^ The n-type contact also incorporated a C_60_ layer deposited by thermal evaporation to enhance electron extraction. Here, an additional SnO_2_ layer was included (which was not present in our previous work) to prevent undesirable reactions between titanium and C_60_, with these reactions occurring at low temperature and in the solid state.^31^ Such reactions have been demonstrated to form amorphous titanium carbide which can result in the passivation of metals against corrosion, thus likely fouling metal electrodes in electronic devices causing increased series resistance.^33^

As can be seen in Figure 1c, there is an overlap between the metal contacts of neighboring grooves at the apex of each groove. This creates a serial electrical connection between the grooves; a feature that–as we show below–allows us to create discrete minimodules that we term “cascades”. In the experiments described below, we have explored cascades composed of either 50 or 362 series-connected grooves; with the number of grooves in a cascade defined by the layout of the pattern initially embossed onto the plastic roll. Note that the substrate was also patterned with so-called “delineation features”. These consist of a set of deeper grooves that separate cascades without producing any photocurrent (as investigated below). We have chosen to emboss cascades containing relatively large numbers of grooves (up to 362), as this allows us to maximize the geometric fill factor - i.e., the relative area of the module that can produce a photocurrent.

We have performed extensive optimization experiments to enhance device efficiency; for the preparation of the substrate, this involved exploring the effect of deposition angle, partial pressure of oxygen in the deposition chamber during reactive depositions (affecting the degree of oxidation), individual layer thicknesses and the use of initial plasma-treatments on the embossed substrate. This optimization process and its effect on device efficiency is summarized in Supporting Information Figure S1. The approximate thicknesses of the various charge-transport and extraction layers in optimized devices are detailed in Table 1 and have been estimated via cross-sectional microscopy as presented later. As a result of the geometry of the deposition process, the deposition angle and the shape of the groove, the thickness of each individual evaporated layer is often found to vary as a function of depth into the groove.

**Table 1:** Approximate Layer Thicknesses in Champion Groove Devices, Estimated from SEM Cross-Sectional Imaging

material	approximate thickness (nm)
Ni	35
NiO_*x*_	25
Ti	55
SnO_2_	25
C_60_	20

The final step in the fabrication process involved the deposition of the perovskite MAPbI_3_ (where MA refers to methylammonium, CH_3_NH_3_^+^) from an acetonitrile (ACN) solvent.^32^ This system was chosen as it could be converted to a perovskite with only short and low-temperature annealing. This was required to minimize possible warping or melting of the plastic substrate.

The perovskite precursor was deposited by slot-die coating. This is a scalable technique that has recently been used to fabricate fully roll-to-roll coated, conventional architecture flexible PSC modules, having a PCE of 11.0%.^34^ In our experiments, the substrate was translated relative to the slot-die head at a web speed of 1 m/min. A typical coating run involved the deposition of perovskite over a 3 m length of substrate, creating around 4000 individual device cascades. The coated substrate was then heated to 85 °C using an in-line oven and finally respooled without any further processing being applied. Here, device efficiency was improved by extensive optimization studies that explored effects such as web speed, ink flow rate, solution concentration, the effect of temperature, and the use of gas quench as summarized in Figure S1.

**Figure 2:**
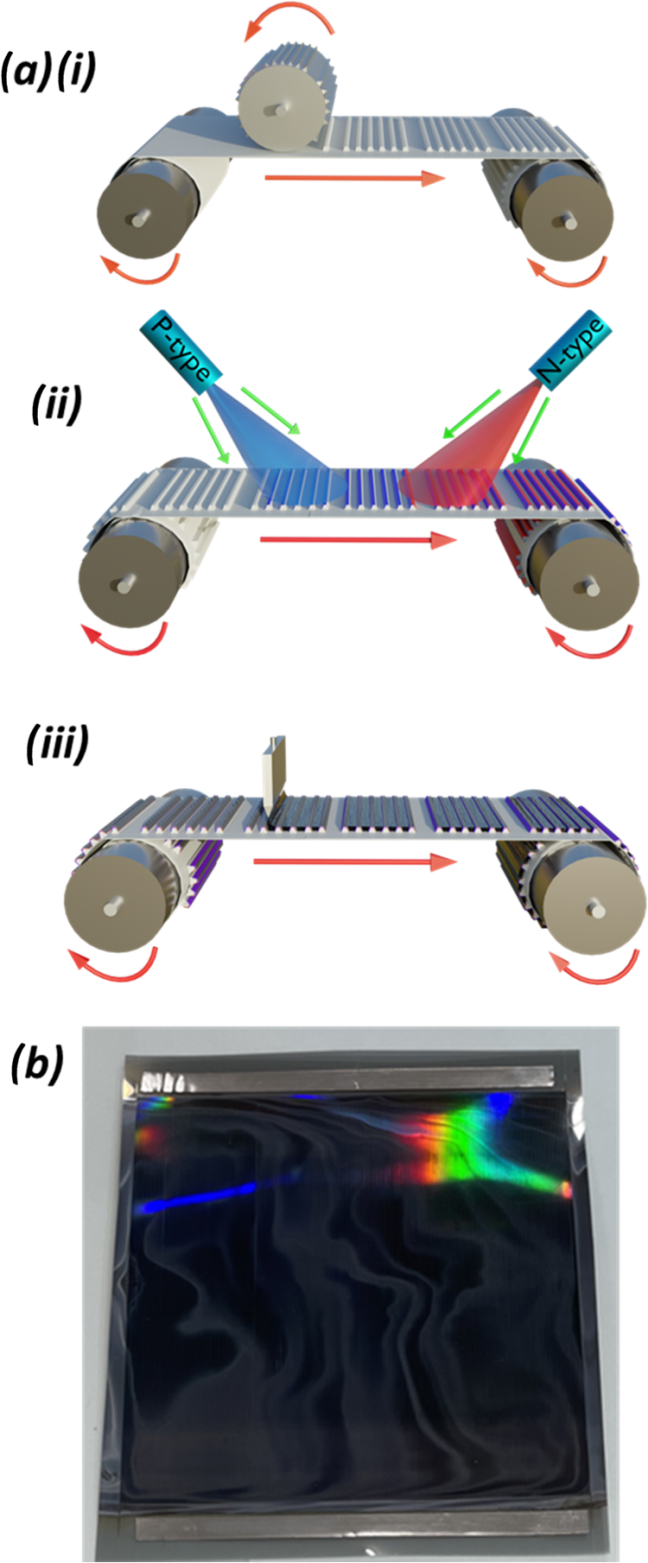
(a) Schematic of roll-to-roll processing of flexible groove-based perovskite solar cells showing roll-to-roll (i) embossing, (ii) evaporation of transport layers, and (iii) slot-die coating of perovskite (groove numbers and dimensions not to scale); (b) photograph of a roughly 10 × 10 cm unit of grooves from which test samples are cut.

The complete fabrication process is shown schematically in Figure 2a. Figure 2b shows an image of part of the coated web; here the grooves that are embossed into the surface are evident via the colorful optical interference of the reflected light. For comparison, structures on smaller sections of the substrate were also fabricated from the same precursor by spin-coating. Owing to the relatively sensitive nature of the embossed polymeric substrate, our perovskite precursor formulation has been optimized around a short, low-temperature anneal. The absorption of a typical MAPbI_3_ film spin-cast on a quartz substrate is shown in Figure S2. In all cases, the resultant devices had the following architecture: Ti\SnO_2_\C_60_\MAPbI_3_/NiO/Ni (see Figure 1d). Grazing incidence wide-angle X-ray Scattering (GIWAXS) measurements (see Figure S3) confirmed the presence of a perovskite structure within the grooves with no large-scale directionality observed. X-ray diffraction (XRD) measurements also demonstrate that the crystalline structure of spin-cast and slot-die coated MAPbI_3_ are very similar (See Figure S4).

Figure 1e shows a band diagram across a single groove device, with data plotted for NiO, SnO_2_, C_60_ and MAPbI_3_. Here, Ultraviolet Photoelectron Spectroscopy (UPS) measurements (recorded using UV HeI photons), allowed us to calculate the valence-band edge from the work-function of the different materials via the measured electron binding energies. By constructing a Tauc plot from optical absorption measurements, we were also able to calculate the optical bandgaps of SnO_2_ and NiO, with a value for MAPbI_3_ taken from Noel et al.^32^ This process is described in more detail in methods with data summarized in Figures S5 to S7. It can be seen in Figure 1e that the conduction band of MAPbI_3_ at −4.0 eV is well matched with the work function of Ti and the conduction bands of SnO_2_ and C_60_, being −4.3, −4.2, and −4.0 eV respectively, suggesting facile electron extraction. We also observe a 0.4 eV difference between the valence band of MAPbI_3_ and the work function of Ni. However, the conduction band offset between MAPbI_3_ and NiO is significantly larger than the valence band offset of MAPbI_3_ and C_60_, implying that NiO is more effective at blocking electrons than C_60_ is at blocking holes.

### Characterizing the Device Structure

**Figure 3:**
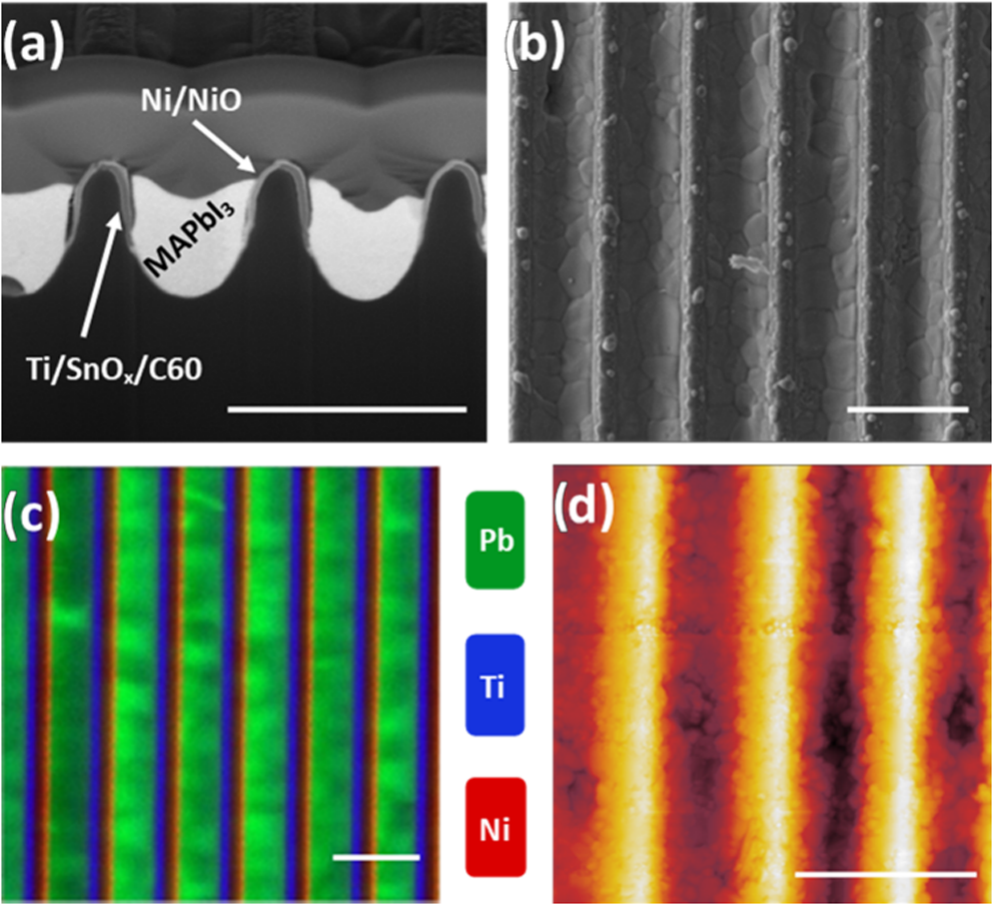
(a) FIB-SEM cross-section of groove device showing multiple grooves in series; (b) top-down SEM of five grooves in series; (c) X-ray fluorescence map of a fully roll-to-roll coated groove cascade with lead, titanium and nickel shown in green, blue and red respectively; (d) AFM of four grooves in series (not matching areas). All scale bars 2 μm.

In order to optimize the efficiency of the groove-based perovskite solar cells, we have explored the crystallinity, topography and chemical identity of the constituent materials. Gaining a full understanding of device morphology over length scales commensurate with charge-diffusion lengths is critical in order to identify structures that may result in performance losses. Figure 3a shows an SEM image recorded in cross-section through a typical series of grooves. For completeness, SEM images of a series of unfilled grooves are shown in Figure S8. As discussed above, grooves were embossed with a “U-shaped” cross-section, which is clearly visible in the cross-sectional SEM. Here, we can individually identify and quantify the thicknesses of the Ti, SnO_2_, C_60_, Ni and NiO layers, as listed in Table 1. It is clear that each wall of the groove is coated with a different series of materials that overlap at the apex of the grooves. However, identifying the chemical composition and distribution along the grooves is impossible from the SEM images alone.

As can be seen in Figure 3a the perovskite (visible as a light-gray region) largely fills the grooves, although a small void is often observed at the bottom of the grooves. We believe that such voids likely result from trapped solvent remaining after precursor deposition or partial dewetting of the ink from the bare substrate at the bottom of the groove. A small amount of perovskite material can be seen at the apex of some grooves in the top-down SEM and AFM images shown in Figure 3b,c respectively. These can be a source of performance loss in the devices as this overburden can create shunting pathways between adjacent grooves, resulting in reduced module performance. We have therefore optimized our coating process to minimize the volume of material deposited at the groove apex; such optimization required control over solution concentration, coating speed and slot-die coating (slot-die head geometry, solution flow rate, environmental conditions, etc.).

Even without perovskite being present, the distribution of the transport layers cannot be elucidated by SEM alone. To explore the distribution of the various metals that form the contacts, we have used nanofocus X-ray Fluorescence (XRF) mapping to characterize devices at various stages in their fabrication. The nanofocus technique involves using an X-ray beam generated by a synchrotron (Diamond Light Source station I14) which is focused to a 50 nm diameter spot. The beam spot was then scanned across the surface with the emitted X-rays dispersed using a spectrometer to determine which elements are present from their characteristic X-ray emission energies. This technique produces high-resolution maps of the relative chemical composition of the film at 50 nm resolution, bypassing the optical diffraction limit.

A typical scanning XRF image of a series of perovskite-filled grooves is shown in Figure 3c, with the distribution of Ni, Ti and Pb plotted using red, blue, and green respectively. Images of each material individually deposited onto the grooves are shown in Figures S9 and S10. We find, as anticipated, the n- and p-type layers are clearly defined, being coated onto opposing walls with no evidence of any contact between these layers at the bottom of the groove (which might otherwise provide shorting pathways). From the image, we determine an approximate separation between n- and p-type contacts at the surface of around 950 nm. Note that the tin signal is generally weaker than that of the other elements and has significant spectral overlap with Ti emission so is not clearly distinguishable (Figure S9), nevertheless, the presence of SnO_2_ can be confirmed from the cross-sectional SEM images. The elemental distribution across the substrate has also been confirmed using EDX SEM as shown in Figures S13 and S14.

The scanning XRF measurements allow us to gain some insight into the structure of the perovskite within the grooves. It is apparent that there is some fluctuation in the magnitude of the lead signal over length scales of around a micron. Interestingly, we also find that the average lead signal varies *between* neighboring grooves; a result that suggests that some grooves have a thicker perovskite layer (i.e., deeper fill into the groove) than others. From the above-discussed SEM images, we find that the surface-level of the perovskite does not vary appreciably across the grooves, suggesting that some of the local variations in the lead and iodine signals may instead result from variations in the size and shape of subsurface voids located at the bottom of the grooves. The origin of the variation of the size and distribution of such voids is currently not understood; they may result from slight differences in the depth or dimensions of the grooves or local variations in the distribution of the charge-extraction contacts which result in enhanced local dewetting. We also find that the lead signal can vary by a factor of 2 across the width of one groove (Figure S11); a result suggesting that the perovskite is thicker toward the nickel-coated walls. We speculate that this may result from improved wetting of the precursor on the NiO surface compared to the opposing C_60-_coated contact. We also find fluctuations in the relative ratio of lead and iodine in the perovskite across the surface (Figure S12) suggesting that certain areas are lead-rich–possibly resulting from local regions of PbI_2_. Interestingly, in samples in which the perovskite precursor was spin-cast, these regions seem to be more localized to specific grooves; an effect that may result from variations in solvent drying dynamics between the two methods. Note however, that we do not find evidence for systematic changes in the stoichiometry of the perovskite across the width of the grooves when deposited roll-to-roll via slot-die coating (Figure S11).

As was evident from the top-down SEM images, we again clearly observe perovskite grain structure in our AFM measurements, as shown in Figure 3d. We can postprocess such images to more clearly identify grain boundaries as shown in Figure S15. From this, we determine that typical grains have a mean size of 114 nm. This compares with the mean grain size of control perovskite films that were spin-cast onto a quartz-glass surface of 105 nm. We note that the average separation between the walls of the grooves (and thus the charge-extraction contacts) is around 950 nm. This distance is greater than the thickness of the perovskite active layer in a conventional-architecture solar cell (which is typically 300–600 nm), and thus we expect that photogenerated charges within each groove will likely have to traverse a number of grain boundaries before being extracted at a device contact. Recombination at grain boundaries may therefore act as a loss mechanism within our devices.

### Charge Separation and Generation of Photocurrent

**Figure 4:**
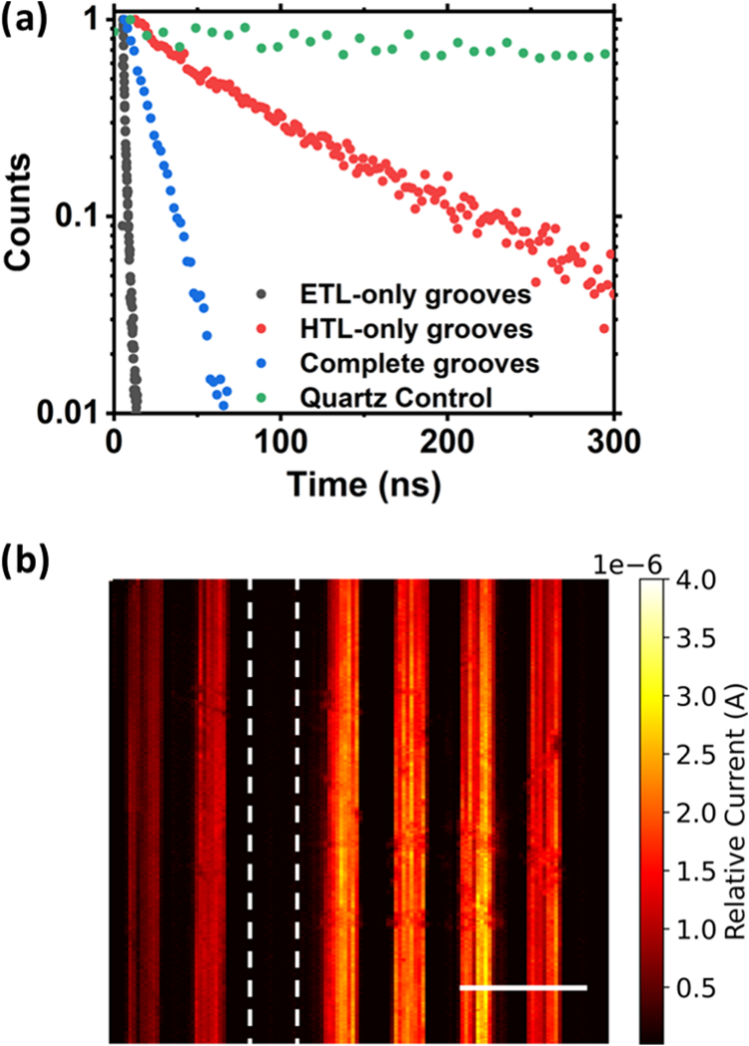
(a) TCSPC decay curves of MAPbI_3_ on a quartz substrate and grooves featuring one or both walls coated, recorded using Time-Correlated Single-Photon Counting. (b) Photocurrent map showing current generated from a series of device cascades. Position of shunted cascade indicated by white dashed lines. Scale bar 500 μm.

Having validated the distribution of the charge transport and contact materials and the polycrystalline nature of the perovskite, we now explore the ability of such devices to generate and extract a photocurrent. Here, we first demonstrate the function of transport materials to act as charge extraction layers via characterizing photoluminescence decay lifetime of the MAPbI_3_ perovskite when spin-coated on a series of different representative substrates using Time-Correlated Single-Photon Counting (TCSPC). This is shown in Figure 4a where we present example decay curves of perovskite deposited into grooves where only one of the walls was coated with a contact material. These substrates are designed to explore the efficiency by which each of the different charge-transport layers are able to extract charge-carriers from the device. For completeness, the photoluminescence decay of the perovskite spin-coated on a quartz substrate is also shown. The data presented in Figure 4a was taken from part of a larger data set with measurements made at a series of different points across each substrate. We show histograms of these decay lifetimes across 10 μm × 10 μm areas in Figure S16, with data also recorded on quartz substrates (Figures S17 and S18). Our analysis of this data set indicates median decay lifetimes of 1.0, 72, and 14 ns for the perovskite deposited on ETL-only (C_60_/SnO_2_), HTL-only (NiO/Ni), and grooves with both walls coated respectively. This compares to the median lifetime of 1252 ns of an identical MAPbI_3_ film spin-coated onto a quartz substrate without any transport layers.

Clearly, therefore, the different transport layers modify charge recombination mechanics in different ways. As the photoluminescence intensity is proportional to the local electron and hole density, we associate a reduction in decay lifetime with an increase in the nonradiative recombination rates. Indeed, we suspect that the SnO_2_/C_60_ ETL interface is characterized by a series of defects which enhance nonradiative recombination. The observed reduction in the emission lifetime of the perovskite in single-carrier devices can therefore be attributed to increased nonradiative recombination around the perovskite/transport layer interface. Interestingly, the observed decay lifetime of the structure containing *both* ETL and HTL contacts is intermediate between those with either ETL or HTL alone. We propose therefore that the built-in field in the full device helps to reduce nonradiative recombination by moving holes away from the ETL/perovskite interface.

As discussed above, the series-connected grooves embossed onto the substrate form modules which we term cascades. Between each adjacent cascade, we also emboss a series of deeper, wider grooves, known collectively as “delineation features”. The added depth of these features ensures that transport layers are not in contact with perovskite and can thus electrically isolate each cascade from those adjacent. Extending these delineation features to cap the ends of a cascade of grooves aids to further direct extraction of electrons and holes in a direction perpendicular to the cascades. This also allows cascades to be connected in parallel simply through the use of conductive tracks at opposite sides of the substrate. This allowed simultaneous connection to be made to either the n- or p-type side of the cascades. A schematic of a typical layout of the cascades allowing their parallel connection is shown in Figure S19.

To evidence photovoltaic activity in parallel connected cascades, we have recorded maps of photocurrent generation following optical excitation at 637 nm using compressive sensing current mapping. In this technique, a series of different pixel patterns having varying spatial frequency are projected at the cascades via an optical microscope.^35,36^ Here, the photocurrent generated by the cascades is recorded for each projected pattern, and by postprocessing the data, it is possible to reconstruct the spatial distribution of photocurrent generated across the surface. A typical image of the photocurrent generated across a series of 50-groove cascades is shown in Figure 4b. Due to the limited resolution of the optical microscope, we do not clearly resolve individual grooves within each cascade. However, we find a relatively uniform photocurrent generated across individual cascades, indicating a comparable level of performance between the individual cascades. Interestingly, we find that one cascade generated no measurable current (white dashed line). This implies the cascade was shunted, which could be caused by poor electrical connection to the cascade during measurement, or by significant shorting pathways from overfilled grooves. Nevertheless, it is clear that the failure of this one cascade does not affect the performance of neighboring cascades, with such parallel connected cascades forming separate PV minimodules.

### Electronic Characterization of Devices

Having explored the physical structure of the groove devices and their ability to generate and extract a photocurrent, we now discuss the performance of our devices and focus our attention on devices fabricated by slot die coating, although devices fabricated by spin coating have very similar performance. For testing purposes, the coated roll shown in Figure 2b was first cut into small sections of around 1.5 cm × 1.5 cm. Each cut section typically contained around 20 cascades, with each cascade comprising 362 serial connected grooves. The act of cutting sections from the substrate surface meant that the cascades were no longer connected in parallel at the edges of the substrate, but instead were electrically isolated from each other by the delineation features. This allowed the JV characteristics of individual cascades to be recorded using a probe station with alignment of the probes at opposite sides of the cascade performed using a microscope. No aperture mask was required in this measurement, as our previous work has shown this is unnecessary when illuminating these devices. Figure S20 shows an image of a section of coated substrate together with a schematic indicating how different areas are selected for study. In our calculation of PCE, we first calculate the active area of each cascade from the known width of the individual grooves (1.5 μm), the number of grooves in each cascade (e.g., 362) and their length as measured using callipers (typically 1.5 cm). This gives a typical active area of around 0.0815 cm^2^. Note, we also assume a geometric fill factor of 82% to account for the area of the cascade in which current is generated, discounting the area at the apex of each groove. When we include this geometric fill factor, we determine a typical active area for each cascade of 0.0689 cm^2^.

Devices were tested under 1 sun using a calibrated AAA solar simulator, with light directed to the grooves through the transparent plastic substrate (i.e., the solar simulator was placed underneath the probe station with light projected upward). Interestingly, we find that a photocurrent could be generated whether the device is illuminated from above or through the substrate, with illumination from beneath resulting in higher recorded efficiencies. This indicates that groove devices can be regarded as a bifacial technology.

**Figure 5:**
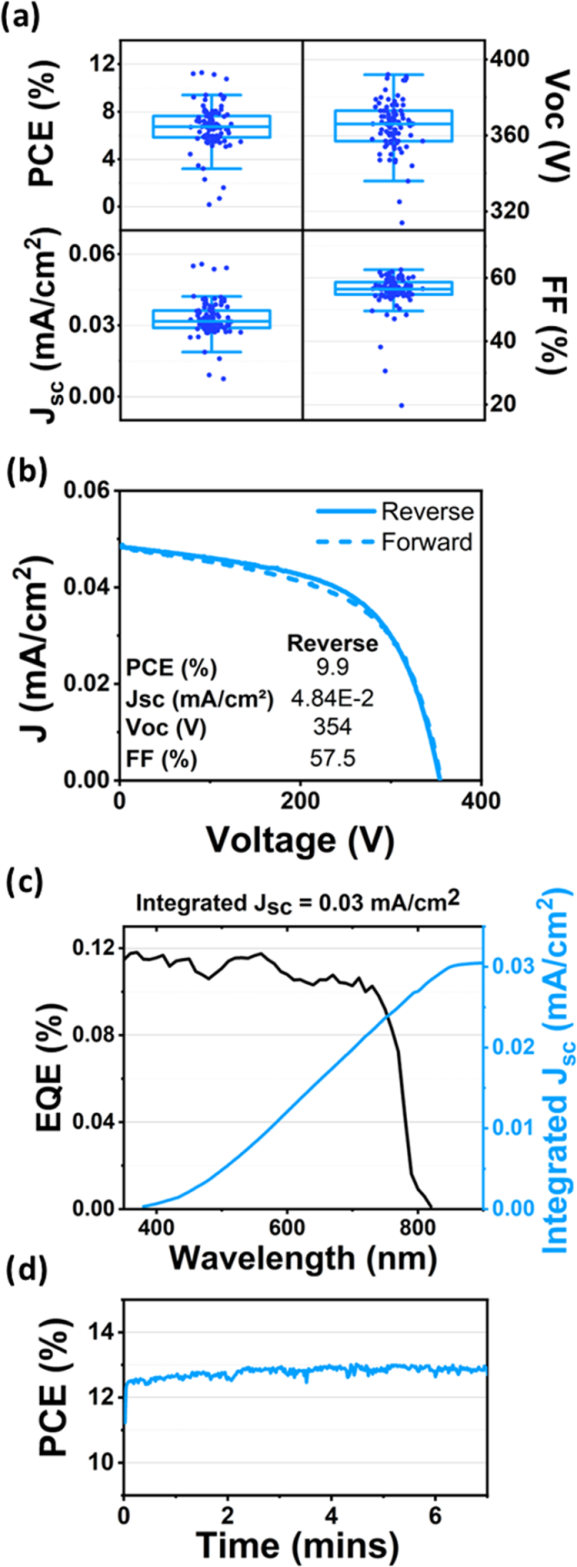
(a) Box plots of performance metrics for fully roll-to-roll evaporated and coated groove devices; (b) example *J*–*V* curve of fully roll-to-roll coated cascade with metrics inset; (c) external quantum efficiency of representative groove device; (d) stabilized efficiency of champion cascade.

Figure 5a is a box plot of device metrics recorded from 23 individual cascades. We find that cascades had mean values of PCE, *J*_sc_, *V*_oc_ and fill factor of 6.7%, 0.033 mA/cm^2^, 359 V and 55.7% respectively. Here the champion cascade had a PCE, *J*_sc_, *V*_oc_ and fill factor of 11.3%, 0.056 mA/cm^2^, 373 V and 54.2% respectively. A *JV* curve of a typical cascade is shown in Figure 5b. As these modules consisted of 362 grooves, short-circuit currents and open circuit voltages can be scaled by this value to represent a comparable value to flat devices. In doing this, we find typical cascades have equivalent *J*_sc_ and *V*_oc_ values of 11.9 mA/cm^2^ and 0.99 V, with champion values of 20.3 mA/cm^2^ and 1.03 V determined. We can in fact compare the performance of groove cascades with that of regular planar architecture perovskite PV devices having a similar composition in terms of layer structure (Figure S21). Here, we find that p–i–n and n–i–p devices with the same transport layers and perovskite with no annealing have PCEs of 11.3 and 16.3% respectively. Note that the efficiency of these controls are similar to the anticipated performance of MAPbI_3_ prepared from an ACN precursor which has not been subject to thermal annealing (around 17%).^29^

Figure 5d plots the stabilized efficiency of a champion cascade (362 grooves) which reaches an efficiency of 12.8% after 7 min of testing. This performance should be compared with our previous work in which we demonstrated a PCE of 7.03% for a single groove, and 2.63% for a mini-module consisting of 16 grooves. The significant increase in performance for these up-scaled grooves results from the extensive optimization experiments performed over a period of 1 year in which around 10,000 individual cascades were fabricated and tested.

In Figure 5c we plot the external quantum efficiency of a series of cascades. It can be seen that as expected the device response falls to zero at the perovskite band-edge around 820 nm. Note the relatively low EQE of the cascades results from the fact that the short-circuit current output from a module is relatively small as discussed above. We note that if we multiply the peak EQE (0.118%) by the number of grooves in the cascade (362), we obtain a peak EQE value of 42.7%; a value in reasonable agreement with what can be expected from a conventional architecture perovskite solar cell. We can also use our EQE measurement to calculate integrated photocurrent across the visible spectrum and determine an integrated short-circuit current to 0.03 mA/cm^2^; a value in good agreement with the average short-circuit current measured from the *JV* studies reported in Figure 5a.

We have also briefly investigated the shelf life stability of spin-coated cascades (see Figure S22). Here, substrates were stored without encapsulation under nitrogen for a period of 37 days and tested periodically. We observed that in 50-groove cascades, PCE increased considerably in the first few days after fabrication, with the champion cascade reaching its peak efficiency of 10.9% (average 9.9%) at day 5. After 14 and 37 days, the efficiency of cascades had stabilized, demonstrating an average PCE of 7.3%. It was found that *V*_oc_ remained relatively constant throughout the testing period, with *J*_sc_ and FF undergoing a variation that accounted for the observed changes in efficiency.

## Discussion

We have shown that back-contact groove-based modules represent a new approach to the high-volume manufacture of perovskite solar cells. Devices are made using a directional vapor-based coating technique applied to an embossed substrate that creates micron-width, electrically separated n- and p-type contacts with high fidelity. Each of the coating and patterning processes used have been individually performed using a roll-to-roll based process, with the MAPbI_3_ perovskite deposited grooves by slot-die coating. We have characterized the structure of our devices using a range of microscopy and spectroscopy techniques. It is apparent that some compositional and ink fill inhomogeneity is present within individual grooves and from groove-to-groove which we believe may result from nonuniform drying dynamics of the precursor ink, or differences in the wettability of the n-and p-type contact materials. Significantly, we often identify the presence of submicron-sized voids at the bottom of the grooves. The effect of such voids is not currently understood, though as charge extraction occurs in a direction parallel to the substrate, such voids may not have any noticeable effect on device performance. This is clearly an open question and is ripe for further studies. We show that the average size of the perovskite grains is smaller than the width of the grooves, indicating that extracted charges likely have to traverse one or more grain boundaries. It is possible that recombination at such grain boundaries is one factor that limits device performance, however it may be possible to reduce such effects via appropriate passivation strategies.

We note that device efficiency is likely to be dependent on the size and shape of the grooves, with narrower grooves expected to reduce the length of charge-carrier extraction pathways and therefore help to suppress charge-carrier recombination. Deeper grooves that are fully filled with perovskite are also expected to enhance light absorption and therefore increase short-circuit current. However, the fabrication of very narrow and deep grooves using embossing techniques is not without its challenges, as is completely filling high aspect-ratio grooves with a perovskite. Future work will address these issues.

It is clear that the primary loss mechanism in the devices studied results from a relatively low fill factor. We suspect that the primary contribution to this effect results from a series resistance created at the electronic contact between adjacent grooves. It is possible that this electronic loss can be mitigated by modifying the structure and layout of the grooves to enhance the contact area between adjacent grooves and thereby improve the fill factor.

Measurements of radiative emission lifetime indicate that decay lifetimes are reduced significantly when the perovskite is placed in contact with the SnO_2_/C_60_ electron extracting contact, compared to the NiO/Ni contact alone. This suggests that there may be a population of defects or trap-states at the interface between the SnO_2_/C_60_ contact and the perovskite that enhance nonradiative recombination. Future work will explore the development of material systems that reduce such nonradiative recombination, with this likely to result in improvements in device module efficiency.

We have used photocurrent mapping techniques to explore the uniformity of photocurrent generated by parallel connected minimodules, each composed of 362 groove cascades. Here we occasionally find that cascades appear to generate no measurable current, indicating that they are at short circuit. The origin of such effects is not understood, though this does not affect adjacent cascades and indicates that significant improvements in overall module efficiency can be expected via improving the homogeneity of the perovskite deposition process.

Finally, further work will investigate the effect of extended exposure to the combined effects of moisture, light and heat. Critically, the demonstration of stable devices will require the development of an encapsulation system appropriate for our device architecture. This is likely to be rather different from the encapsulation processes that are used to protect regular architecture perovskite devices in which the active layer is located beneath charge-transporting layers and metal contacts, which both provide some degree of environmental protection. As the perovskite in groove devices is exposed, care will be required in choosing materials appropriate for encapsulation to avoid unwanted chemical reactions.

## Conclusions

In summary, we have explored the upscaling via slot-die coating of a novel MAPbI_3_ perovskite back-contact solar-cell technology and have shown that cascades of serially connected grooves can be created having a stabilized efficiency of up to 12.8%. We believe this demonstration of flexible back-contact perovskite solar cell modules manufactured by fully scalable processes that only contains low-cost Earth-abundant materials represents a very promising step toward the commercialization of this technology.

## Supporting Information Available

Methods and materials, supporting data (GIWAXS, UV–vis, XRD, Tauc plots, SEM, XRF, AFM, TCSPC, and device layout schematics) (PDF)

## References

[ref1] Kojima A.; Teshima K.; Shirai Y.; Miyasaka T. Organometal Halide Perovskites as Visible-Light Sensitizers for Photovoltaic Cells. J. Am. Chem. Soc. 2009, 131 (17), 6050–6051. 10.1021/ja809598r. 19366264

[ref2] Hutter E. M.; Eperon G. E.; Stranks S. D.; Savenije T. J. Charge Carriers in Planar and Meso-Structured Organic–Inorganic Perovskites: Mobilities, Lifetimes, and Concentrations of Trap States. J. Phys. Chem. Lett. 2015, 6 (15), 3082–3090. 10.1021/acs.jpclett.5b01361. 26267206

[ref3] Xing G.; Mathews N.; Lim S. S.; Yantara N.; Liu X.; Sabba D.; Grätzel M.; Mhaisalkar S.; Sum T. C. Low-temperature solution-processed wavelength-tunable perovskites for lasing. Nat. Mater. 2014, 13 (5), 476–480. 10.1038/nmat3911. 24633346

[ref4] Brenner T. M.; Egger D. A.; Kronik L.; Hodes G.; Cahen D. Hybrid organic—inorganic perovskites: low-cost semiconductors with intriguing charge-transport properties. Nat. Rev. Mater. 2016, 1 (1), 15007 10.1038/natrevmats.2015.7.

[ref5] Chen B.; Baek S.-W.; Hou Y.; Aydin E.; De Bastiani M.; Scheffel B.; Proppe A.; Huang Z.; Wei M.; Wang Y.-K.; et al. Enhanced optical path and electron diffusion length enable high-efficiency perovskite tandems. Nat. Commun. 2020, 11 (1), 1257 10.1038/s41467-020-15077-3. 32152324 PMC7062737

[ref6] Minami T. Present status of transparent conducting oxide thin-film development for Indium-Tin-Oxide (ITO) substitutes. Thin Solid Films 2008, 516 (17), 5822–5828. 10.1016/j.tsf.2007.10.063.

[ref7] Kawajiri K.; Tahara K.; Uemiya S. Lifecycle assessment of critical material substitution: Indium tin oxide and aluminum zinc oxide in transparent electrodes. Resour., Environ. Sustainability 2022, 7, 100047 10.1016/j.resenv.2022.100047.

[ref8] Nassar N. T.; Graedel T. E.; Harper E. M. By-product metals are technologically essential but have problematic supply. Sci. Adv. 2015, 1 (3), e1400180 10.1126/sciadv.1400180. 26601159 PMC4640630

[ref9] Wagner L.; Suo J.; Yang B.; Bogachuk D.; Gervais E.; Pietzcker R.; Gassmann A.; Goldschmidt J. C. The Resource Demand of Terawatt-Scale Perovskite Tandem Photovoltaics. Elsevier BV: 2023.

[ref10] Lokanc M.; Eggert R.; Redlinger M. The Availability of Indium: The Present, Medium Term, and Long Term; National Renewable Energy Laboratory, 2015.

[ref11] Tuyen L. T. C.; Jian S.-R.; Tien N. T.; Le P. H. Nanomechanical and Material Properties of Fluorine-Doped Tin Oxide Thin Films Prepared by Ultrasonic Spray Pyrolysis: Effects of F-Doping. Materials 2019, 12 (10), 1665 10.3390/ma12101665. 31121861 PMC6566963

[ref12] Lammert M. D.; Schwartz R. J. The interdigitated back contact solar cell: A silicon solar cell for use in concentrated sunlight. IEEE Trans. Electron Devices 1977, 24 (4), 337–342. 10.1109/T-ED.1977.18738.

[ref13] Jumabekov A. N.; Della Gaspera E.; Xu Z. Q.; Chesman A. S. R.; Van Embden J.; Bonke S. A.; Bao Q.; Vak D.; Bach U. Back-contacted hybrid organic–inorganic perovskite solar cells. J. Mater. Chem. C 2016, 4 (15), 3125–3130. 10.1039/C6TC00681G.

[ref14] Song Y.; Bi W.; Wang A.; Liu X.; Kang Y.; Dong Q. Efficient lateral-structure perovskite single crystal solar cells with high operational stability. Nat. Commun. 2020, 11 (1), 274 10.1038/s41467-019-13998-2. 31937785 PMC6959261

[ref15] Hou Q.; Bacal D.; Jumabekov A. N.; Li W.; Wang Z.; Lin X.; Ng S. H.; Tan B.; Bao Q.; Chesman A. S. R.; et al. Back-contact perovskite solar cells with honeycomb-like charge collecting electrodes. Nano Energy 2018, 50, 710–716. 10.1016/j.nanoen.2018.06.006.

[ref16] Wong-Stringer M.; Routledge T. J.; McArdle T.; Wood C. J.; Game O. S.; Smith J. A.; Bishop J. E.; Vaenas N.; Coles D. M.; Buckley A. R.; Lidzey D. G. A flexible back-contact perovskite solar micro-module. Energ Environ. Sci. 2019, 12 (6), 1928–1937. 10.1039/C8EE03517B.

[ref17] Zhao B.; Gillan L. V.; Scully A. D.; Chesman A. S. R.; Tan B.; Lin X.; Liu J.; Rietwyk K. J.; Deng S.; Bailey C.; et al. Enhanced Carrier Diffusion Enables Efficient Back-Contact Perovskite Photovoltaics. Angew. Chem., Int. Ed. 2023, 62 (27), e202218174 10.1002/anie.202218174. 36951117

[ref18] Lim J.; Kober-Czerny M.; Lin Y.-H.; Ball J. M.; Sakai N.; Duijnstee E. A.; Hong M. J.; Labram J. G.; Wenger B.; Snaith H. J. Long-range charge carrier mobility in metal halide perovskite thin-films and single crystals via transient photo-conductivity. Nat. Commun. 2022, 13 (1), 4201 10.1038/s41467-022-31569-w. 35859149 PMC9300620

[ref19] Xing G.; Mathews N.; Sun S.; Lim S. S.; Lam Y. M.; Grätzel M.; Mhaisalkar S.; Sum T. C. Long-Range Balanced Electron- and Hole-Transport Lengths in Organic-Inorganic CH 3 NH 3 PbI 3. Science 2013, 342 (6156), 344–347. 10.1126/science.1243167. 24136965

[ref20] Dong Q.; Fang Y.; Shao Y.; Mulligan P.; Qiu J.; Cao L.; Huang J. Electron-hole diffusion lengths > 175 μm in solution-grown CH 3 NH 3 PbI 3 single crystals. Science 2015, 347 (6225), 967–970. 10.1126/science.aaa5760. 25636799

[ref21] Stranks S. D.; Eperon G. E.; Grancini G.; Menelaou C.; Alcocer M. J. P.; Leijtens T.; Herz L. M.; Petrozza A.; Snaith H. J. Electron-Hole Diffusion Lengths Exceeding 1 Micrometer in an Organometal Trihalide Perovskite Absorber. Science 2013, 342 (6156), 341–344. 10.1126/science.1243982. 24136964

[ref22] Prince K. J.; Nardone M.; Dunfield S. P.; Teeter G.; Mirzokarimov M.; Warren E. L.; Moore D. T.; Berry J. J.; Wolden C. A.; Wheeler L. M. Complementary interface formation toward high-efficiency all-back-contact perovskite solar cells. Cell Rep. Phys. Sci. 2021, 2 (3), 100363 10.1016/j.xcrp.2021.100363.

[ref23] Prince K. J.; Muzzillo C. P.; Mirzokarimov M.; Wolden C. A.; Wheeler L. M. All-Back-Contact Perovskite Solar Cells Using Cracked Film Lithography. ACS Appl. Energy Mater. 2022, 5 (8), 9273–9279. 10.1021/acsaem.2c01298.

[ref24] Deng S.; Tan B.; Chesman A. S. R.; Lu J.; McMeekin D. P.; Ou Q.; Scully A. D.; Raga S. R.; Rietwyk K. J.; Weissbach A.; et al. Back-contact perovskite solar cell fabrication via microsphere lithography. Nano Energy. 2022, 102, 107695 10.1016/j.nanoen.2022.107695.

[ref25] Parkhomenko H. P.; Mangrulkar M.; Jumabekov A. N. Slot-Die-Coated Active Layer for Printed Flexible Back-Contact Perovskite Solar Cells. Coatings 2023, 13 (3), 550 10.3390/coatings13030550.

[ref26] Ščajev P.; Miasojedovas S.; Juršėnas S. A carrier density dependent diffusion coefficient, recombination rate and diffusion length in MAPbI3 and MAPbBr3 crystals measured under one- and two-photon excitations. J. Mater. Chem. C 2020, 8, 10290–10301. 10.1039/D0TC02283G.

[ref27] Milot R. L.; Eperon G. E.; Snaith H. J.; Johnston M. B.; Herz L. M. Temperature-Dependent Charge-Carrier Dynamics in CH3NH3PbI3 Perovskite Thin Films. Adv. Funct. Mater. 2015, 25, 6218–6227. 10.1002/adfm.201502340.

[ref28] Adhyaksa G. W. P.; Veldhuizen L. W.; Kuang Y.; Brittman S.; Schropp R. E. I.; Garnett E. C. Carrier Diffusion Lengths in Hybrid Perovskites: Processing, Composition, Aging, and Surface Passivation Effects. Chem. Mater. 2016, 28 (15), 5259–5263. 10.1021/acs.chemmater.6b00466.

[ref29] Blackburn D.; Routledge T. J.; O’Kane M.; Cassella E. J.; Game O. S.; Catley T. E.; Wood C. J.; McArdle T.; Lidzey D. G. Low-Temperature, Scalable, Reactive Deposition of Tin Oxide for Perovskite Solar Cells. Solar RRL 2022, 6, 2200263 10.1002/solr.202270081.

[ref30] Routledge T. J.; Wong-Stringer M.; Game O. S.; Smith J. A.; Bishop J. E.; Vaenas N.; Freestone B. G.; Coles D. M.; McArdle T.; Buckley A. R. Low-temperature, high-speed reactive deposition of metal oxides for perovskite solar cells. J. Mater. Chem. A 2019, 7 (5), 2283–2290. 10.1039/c8ta10827g.

[ref31] Wang W. H.; Wang W. K. Interactions between the interface of titanium and fullerene. J. Appl. Phys. 1996, 79 (1), 149–152. 10.1063/1.360922.

[ref32] Noel N. K.; Habisreutinger S. N.; Wenger B.; Klug M. T.; Hörantner M. T.; Johnston M. B.; Nicholas R. J.; Moore D. T.; Snaith H. J. A low viscosity, low boiling point, clean solvent system for the rapid crystallisation of highly specular perovskite films. Energy Environ. Sci. 2017, 10 (1), 145–152. 10.1039/C6EE02373H.

[ref33] Alloca C. M.; Williams W. S.; Kaloyeros A. E. Electrochemical Characteristics of Amorphous Titanium Carbide Films Produced by Low-Temperature Metal-Organic Chemical Vapor Deposition (MOCVD). J. Electrochem. Soc. 1987, 134, 3170–3175. 10.1149/1.2100364.

[ref34] Weerasinghe H. C.; Macadam N.; Kim J. E.; et al. The first demonstration of entirely roll-to-roll fabricated perovskite solar cell modules under ambient room conditions. Nat. Commun. 2024, 15, 1656 10.1038/s41467-024-46016-1. 38472219 PMC10933357

[ref35] Koutsourakis G.; Blakesley J. C.; Castro F. A. Signal Amplification Gains of Compressive Sampling for Photocurrent Response Mapping of Optoelectronic Devices. Sensors 2019, 19 (13), 2870 10.3390/s19132870. 31261641 PMC6650843

[ref36] Koutsourakis G.; Thompson A.; Blakesley J. C. Toward Megapixel Resolution Compressed Sensing Current Mapping of Photovoltaic Devices Using Digital Light Processing. Solar RRL 2022, 6 (5), 2100467 10.1002/solr.202100467.

