# Supplementary material for: Back-Contact Perovskite Solar Cell Modules Fabricated via Roll-to-Roll Slot-Die Coating: Scale-Up toward Manufacturing
Source: ACS Appl Energy Mater. 2025 Feb 18;8(4):2219–28. doi: 10.1021/acsaem.4c02734 (PMC11863245; doi:10.1021/acsaem.4c02734)
Supplement: Supplementary file 1 — ae4c02734_si_001.pdf [file ae4c02734_si_001.pdf]

Supplementary Information

Back-contact Perovskite Solar Cell Modules Fabricated via Roll-to-Roll Slot-die Coating: Scale-up Towards Manufacture

Dominic Blackburn[1], Nathan S. Hill[2*], Christopher J. Wood[2], Tamilselvan Velusamy[2], Balder A. Nieto-Díaz[2], Caitlin Woolley[2], Andy Brown[2], Loukas Zampelis[2], Trevor McArdle[2], Molly Worth[3], Timothy Thornber[1], Ibrahim Albariqi[1, 4], Rachel C. Kilbride[5], Tingxiang Yang[6], C. Neil Hunter[6], Graham J. Leggett[5], George Koutsourakis[7], James C. Blakesley[7], Fernando A. Castro[7], David Beynon[3], Trystan M. Watson[3], Dumitru Sirbu[2] and David G. Lidzey[1*]

[1] Department of Physics and Astronomy, Hicks Building, Hounsfield Road, Sheffield S3 7RH, United Kingdom

[2] Power Roll Ltd, Jade Business Park, Spring Road, Seaham, SR7 9DR, United Kingdom

[3] Department of Chemistry, Dainton Building, University of Sheffield, Brook Hill, Sheffield, S3 7HF, United Kingdom

[4]School of Biosciences, University of Sheffield, Sheffield S10 2TN, United Kingdom

[5]SPECIFIC, Swansea University, Bay Campus, Fabian Way, Swansea SA1 8EN, United Kingdom

[6]National Physical Laboratory, Hampton Road, Teddington, TW11 0LW, United Kingdom

[7]Physics Department, Faculty of Science, Al-Baha University, Alaqiq, 65779-7738, Kingdom of Saudi Arabia

*d.g.lidzey@sheffield.ac.uk, nathan.hill@powerroll.solar

Methods

Precursor solutions and materials Lead (II) Iodide (99.99%, trace metals basis) and Methylamine solution (ca. 9% in acetonitrile) were purchased from TCI Chemicals. Methylammonium iodide (>99.99%) was purchased from Greatcell Solar Materials. C_{60} was purchased from Ossila Ltd. Starting materials of nickel, titanium and tin were purchased from Testbourn

Groove embossing Groove substrates were prepared using several steps typical of the holographic industry, with the whole process involving several commercial partners. The overall process involved origination of the cascade patterns via photolithography, mastering via Ni electroplating and embossing via roll-to-roll UV casting. These are described in more detail below.

Origination A photoresist was spin cast onto glass and then patterned glass using greyscale photo lithography at IGI-Himax, resulting in product generation photo-resist masters.

Mastering The photoresist masters underwent several replication steps using Ni electroplating at 3D AG or Temicon GmbH. The photoresist master was first prepared via sputter coating of Ni/V or Ag chemical wetting. This was then electroplated with Ni resulting in a tool generation master. This step was repeated on the master resulting in several product generation sub-masters, creating several tool generation shims. The tool generation shims were laser welded onto a cylinder referred to as a sleeve. Blank 'mirror' shims were used to ensure the sleeve met the size requirements for the R2R embossing process.

Embossing The material was R2R embossed at BASF Coatings GmbH using UV-casting. The sleeve was mounted onto an air cylinder and loaded into the embossing equipment. A UV-curable resin was deposited onto the selected PET web material via slot die coating. The sleeve then embossed the pattern into the resin which is then UV cured. This process was typically carried out on substrate lengths of 300 m.

Deposition of charge-transport materials Charge transport materials were deposited using an Angstrom vacuum coater with e-beam and thermal sources inside an Inert PureLab HE 6GB glove box. Deposition of materials occurred Roll-to-Roll at rates between 4 Å/s – 15 Å/s and web speeds from 5 mm/s – 15 mm/s. Materials were deposited at glancing angles onto the respective groove walls, hole transport materials were deposited at positive angles between 48° and 62° and electron transport materials were deposited at negative angles between -62° and -47°

Coating perovskite precursor solutions A 0.5 M $MAPbI_3$ with a 5% Pb excess was fabricated by adding a 9% methylamine solution in acetonitrile to the desired weight of MAI and PbI_2. This precursor solution was based on the formation first developed by Noel et al [7] and included additional process additives [8, 9, 10, 11, 12]. The solution was dissolved at ambient temperatures with stirring. Roll-to-Roll slot die coating was performed on a Coatema Machinery GmbH Basecoater. The ink was loaded into a syringe pump and passed through a 45 µm PTFE hydrophobic filter at a pump speed of 0.65 mL/minute. The embossed web was passed under the slot-die head at an approximate hight of 100 µm at a web speed of 1 m/min. Immediately after printing, the web passed through a set of fan circulated ovens typically set to 85 °C.

Synthesis of Conventional Architecture Devices Pre-patterned 8-pixel, ITO-coated glass substrates (20 Ω/sq) were cleaned by sonication first in dilute Hellmanex, deionised water and IPA for 15 minutes each. Substrates were UV-ozone treated for 20 minutes

n-i-p devices After cleaning, substrates were mounted in an evaporation mask and placed in an electron-beam evaporation chamber, which was pumped down to around 4E-6 mbar. 25 nm of SnOx was evaporated onto the substrates at a rate of 1.75 Å/s. During deposition, oxygen was fed into the chamber to maintain a pressure of 9E-4 mbar. Substrates were removed from the evaporator and subjected to 20 minutes UV-ozone treatment. 0.5 M $MAPbI_3$ (MAI, >99.9%, Ossila) (PbI_2, 99.99%, TCI) in a methylamine solution in acetonitrile (9% solution, TCI) was spin coated onto the substrates at 3000 rpm for 30s. No annealing was employed. A spiro-OMeTAD HTL (Ossila, 86 mg/ml in chlorobenzene) solution containing dopants (11 µL of 300mg/ml FK209 Co (III) TFSI in acetonitrile, 20 µL of 500 mg/ml Li-TFSI in acetonitrile and 34 µL tert-butylpyridine) was spin coated dynamically onto the perovskite at 4000rpm for 30s. Devices were patterned with a razor blade, followed by thermal evaporation of 75 nm gold at a rate of 0.1-1.2 Å/s.

p-i-n devices After cleaning, substrates were mounted in an evaporation mask and placed in an electron-beam evaporation chamber which was pumped down to around 4E-6 mbar. 10 nm of NiOx was evaporated onto the substrate at a rate of 1 Å/s. During deposition, oxygen was fed into the chamber to maintain a pressure of 1E-4 mbar. Substrates were removed from the evaporator and perovskite spin coated as above. Devices were then patterned with a razor blade and transferred to a thermal evaporator. An electron-extracting contact consisting of 23 nm C_{60}, 8 nm BCP and 85 nm silver were then sequentially deposited onto the surface to complete the device.

Characterisation of cascades Connection to individual cascades was made using a micro-probe station and a microscope. Here, probe pins were used to contact the unpatterned area adjacent to each side

of the cascade. A calibrated AM1.5 Ossila solar simulator was then placed underneath the probe station, and the device was illuminated through the back substrate. The current generated was then recorded as the voltage was scanned using a Keithley source measure unit. No spectral mismatch factor was applied. Here, the total area of the cascade was estimated from the product of the cascade width, with the width of the cut strip (as measured using a Vernier).

Characterisation of conventional architecture devices. Devices were tested in air using a Newport 92251A-1000 solar simulator calibrated against silicon reference cell to an intensity of 100 mW/cm^2 and devices were illuminated through a 0.02365 cm^2 aperture mask. A Keithley 237 source meter unit was used to sweep applied bias from 0 to 1.2 V and back at a rate of 0.4 V/s.

External quantum efficiency Measurements were made on parallel connected cascades. Here, crocodile clips were used to connect to the opposite sides of a series of cascades on a coated section of substrate. Data was recorded over a wavelength range of 300-900 nm using a Newport QuantX-300 Quantum Efficiency Measurement System. The system was equipped with a 100W Xenon arc lamp chopped at 25 Hz and focused through an Oriel Monochromator (CS130B).

FIB-SEM Samples were coated with a thin layer (10 - 20 nm) of carbon before being loaded into a Tescan Amber FIB-SEM. Top view images were recorded using a beam voltage and current of 2 keV and 100 pA respectively. To create the cross-section images, a carbon (C) or platinum (Pt) protective strip was first deposited over the region of interest using an ion beam at 5 keV and 20 pA for 10 mins, which was then increased to 30 keV and 150 pA until the desired thickness film had been achieved (>1 µm). A trench was then milled at 30 keV and 2 nA. The face to be imaged was then etched using an ion beam at 30 keV 250 pA. The stage was then tilted to 45° for imaging. Here a stretch filter was used to compensate for the tilt and allow a direct view of the etched surface to be recorded.

EDX SEM A field emission scanning electron microscope (FEI Nova NanoSEM 450, ThermoFisher) equipped with an energy-dispersive X-ray (EDX) silicon drift detector (Oxford Instruments X-MaxN 80, Oxford Instruments) was used to collect EDX data and complementary backscatter electron images. For all measurements a working distance of roughly 5 mm was used. A range of accelerating voltages from 2 keV to 5 keV was used depending on the sample composition to minimise substrate decomposition where possible. Spectral maps were collected at a resolution of 2048 x 1408 pixels per scan, over an area of around 115 µm^2. For each pixel, the electron beam dwell time was 300 ns, with the processing time set to 4 s. Two frames were recorded per scan. All data were processed using Aztec (Oxford Instruments) software, with the binning factor set to 2.

AFM Samples were scanned using a Dimension 3100 AFM (Veeco) in Tapping Mode using Scout 350 HAR probes (NuNano, <15° full cone angle over the last 1 um of the tip) with an aluminium backside coating. The long axis of the grooves on the sample was aligned to the long axis of the cantilever and the fast scan axis was perpendicular to the groove axis. This ensured that the 10° tilt of the AFM cantilever did not cause fouling of the front side of the tip against the sidewalls of the grooves. The cantilever was tuned to a free amplitude of approximately 30 nm and scans were acquired with an amplitude setpoint of 50-65% at a line rate of 0.1 Hz. It was necessary to electrically ground the sample coated with perovskite to eliminate long range electrostatic forces. Images were levelled using a three-point level from the flat sections between grooves. Grain structure was segmented by masking off the flat sections then marking grains by watershed. All processing was carried out in Gwyddion Version 2.64.

GIWAXS measurements were performed using a Xenocs Xeuss 2.0 SAXS/WAXS beamline equipped with a liquid Ga MetalJet X-ray source (Excillum) which produces X-rays with an energy of 9.24 keV. The X-ray beam was collimated in "high-flux" mode (S1 = S2 = 1.2 mm, S3 = S4 = 0.8 mm) and directed at the groove surface at an angle of incidence of 0.3°. Scattered X-rays were detected using a Dectris Pilatus 1M pixel detector normal to the direct beam and positioned ~300 mm from the sample. The sample to detector distance was calibrated using a silver behenate standard in transmission geometry. During measurement, the entire flight path including the sample chamber and collimation tubes were held under vacuum to reduce background air scatter. Data was corrected, reduced and reshaped using a GIXSGUI Matlab toolbox [1]. 1D azimuthally integrated intensity profiles were performed across the full azimuthal and scattering vector ranges.

X-ray Fluorescence Mapping X-ray fluorescence (XRF) imaging was performed at the I14 Hard X-ray Nanoprobe [2]. Here, the XRF signal was collected in backscatter geometry using a 4 element Si drift detector, with the sample to detector distance set to 17 mm. A beam energy of between 6 and 14.5 keV was used in experiments, with the energy of the beam set at approximately 1.5 keV higher than the elemental emission line of interest. The beam size was determined as approximately 50 x 50 nm, and was focused using KB mirrors. Samples were cut to size and assembled into standard beamline holders. Each sample was fly scanned through the focussed X-ray beam, with dwell times ranging between 0.015 s and 0.1 s. Individual elemental maps were generated by summing the total intensity in a 200 eV window around the characteristic X-ray fluorescence line of each element (e.g. Pb-Kα) in the XRF spectra at each pixel.

Fluorescence lifetime measurements The lifetime kinetics were recorded using a home-built Fluorescence lifetime imaging microscope (FLIM) equipped with a spectrometer (Princeton Instruments, Acton SP2558), electron-multiplying charge-coupled device (CCD) camera (Princeton Instruments, ProEM 512), and a single-photon hybrid photodetector (Becker & Hickl, HPM-100-50). A 485 nm pulsed laser (PicoQuant, LDH-D-C-485) with a repetition rate of 50 MHz was applied as light source. Fluorescence emission detection was filtered through a 594 nm long-pass filter (Semrock, BLP01-594R-25). The laser beam was focused on the sample surface to a diffraction limited spot using a 20x/0.5 HD air objective (ZEISS, EC Epiplan-NEOFLUAR) for control samples, and a 100x/1.30 oil objective (Olympus UPlanFLN) for groove samples. The modulation of the laser was synchronized with a time-correlated single-photon counting module (Becker & Hickl, SPC-150). The instrument response function (IRF) of the system is ~130 ps, and the convolution of the decay curves with the IRF was accounted for during fitting. The data was processed with FLIMfit 5.1.1 software.

Photocurrent mapping Current mapping microscopy was realised using an optical system based on [4-6], with an integrated digital micromirror device (DMD) of 1920 x 1080 resolution, with an additional tube lens and microscope objective lens (10×). Here, devices were excited using a 637 nm (50 mW) laser source, with the excitation power used at the sample plane being ~ 5 mW. The photocurrent generated by the device was detected using a 2401 Keithley sourcemeter. We did not control sample temperature but the lab atmosphere was stable around 20°C. Although the measured sample area was preconditioned at J_{sc} conditions for 10 minutes before measurements, the samples were not particularly stable during current mapping microscopy; a factor which affected reconstruction results and resulted in an increased noise background.

Ultra-violet photoelectron spectroscopy (UPS) UPS was carried out on a Kratos Axis Supra XPS Spectrometer using a He(I) plasma source ($h\nu = 21.22eV$). A 55 μm aperture was used to limit area of analysis and spectra collected with a pass energy of 10 eV and step size of 0.1 eV. A bias of 9.0V was applied to samples and the integral charge neutraliser was disabled. The secondary electron cutoff and valence band maximum are estimated at the x-axis intercept.

Ultraviolet-visible (UV-Vis) transmission spectra Transmission ($T(\lambda)$) spectra were obtained using a Perkin Elmer Lambda 9 UV-Vis-NIR spectrometer with a starting wavelength of 800 nm and end wavelength of 250 nm with a step size set to 1 nm. Tauc plots were constructed using $h\nu = \frac{h\nu}{\lambda}$ and $(\alpha h\nu)^{1/2} = (2.303\ A\ h\nu)^{1/2}$ where A is the measured absorption, with $A = 2 - \log{(\%T)}$. The energy gap (Eg) of each material was determined from the Tauc plot by determining the intercept of the absorption band-edge with the energy-axis (x-axis).

Construction of a band-diagram The valence band edge relative to the Fermi level is found from the UPS spectra calculating the gradient and x-intercept of the measured electron binding energy (see data for MAPI in Figure S1). To find the work function, the binding energy (*B.E.*) x-axis is first converted to kinetic energy (*K.E.*) using $K.E. = 21.22eV - B.E.$ The position of the work function is then found by taking the gradient and x-intercept of the kinetic energy at the large shoulder of the plot between 0 and 5 eV. The band diagram is calculated relative to the vacuum level being 0eV.

To find the valence band position relative to the vacuum level, the position of the Fermi level (E_f) is first found by using $Ef = Evac - Work\ Function$, where $Evac = 0\ eV$. The valence band (*VB*) position is then calculated by subtracting the HeI extracted valence band edge relative to the Fermi level using $VB = Ef - Evb$. The conduction band (*CB*) is calculated by adding the valence band (VB) energy relative to the vacuum level to the energy-gap (*Eg*) using $CB = VB + Eg$. This value is given a negative value in the band diagram.

References

[1] Jiang Z. et al. J. Appl. Cryst. **48**, 917-926 (2015)

[2] Quinn PD, Alianelli L, Gomez-Gonzalez M, Mahoney D, Cacho-Nerin F, Peach A, Parker JE. J. Synchrotron Radiat. **28**, 1006-1013 (2021)

[3] Nečas D, Klapetek P, Central European Journal of Physics **10**, 181–188 (2012)

[4] G. Koutsourakis, A. Thompson, and J. C. Blakesley, Sol. RRL, **6**, 2100467 (2022)

[5] L. J. Hornbeck, MRS Bull., **26**, 325–327 (2001)

[6] G. Koutsourakis, M. Cashmore, S. R. G. Hall, M. Bliss, T. R. Betts, and R. Gottschalg, IEEE J. Photovoltaics, **7**, 486–492 (2017)

[7] N. K. Noel, S. N. Habisreutinger, B. Wenger, M. T. Klug, M. T. Hörantner, M. B. Johnston, R. J. Nicholas, D. T. Moore and H. J. Snaith, *Energy & Environmental Science*, 2017, **10,** 145–152.

[8] Yan, S.X.; Han, C.B.; Huang, J.; Chen, Y.; Zhang, X.; Chen, X.; Zhang, Y.; Yan, H. *Nanomaterials* 2021, **11**, 473

[9] M.-H. Li, H.-H. Yeh, Y.-H. Chiang, U. Jeng, C.-J. Su, H.-W. Shiu, Y.-J. Hsu, N. Kosugi, T. Ohigashi, Y.-A. Chen, P.-S. Shen, P. Chen, T.-F. Guo, *Adv. Mater*. 2018, **30**, 1801401

[10] Li, Y., Li, H., Tian, L. et al. *J Mater Sci: Mater Electron* 2020, **31**, 12301–12308

[11] Pan, J., Mu, C., Li, Q., Li, W., Ma, D. and Xu, D. (2016), Room-Temperature, *Adv. Mater*., **28,** 8309-8314.

[12] Li, G., Zhang, T., Zhao, Y., *J. Mater. Chem. A*, 2015, **3**, 19674

Supplementary figures

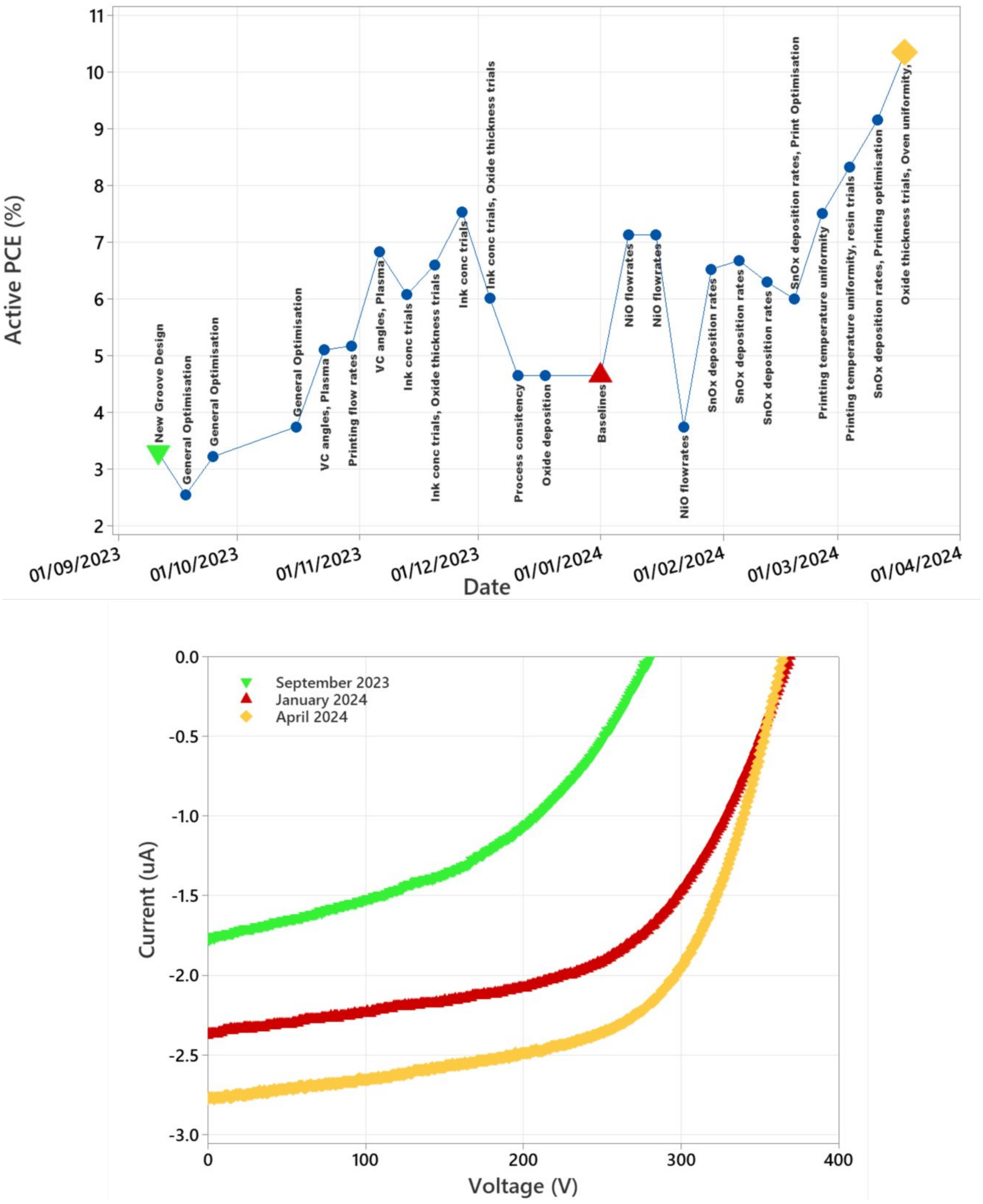

Figure S1: Upper panel shows a timeline of groove-module device efficiency optimisation detailing experimental activity. The lower figure shows example JV scans recorded at different time points during optimisation, with these corresponding to the green, red, and yellow symbols shown in the upper panel.

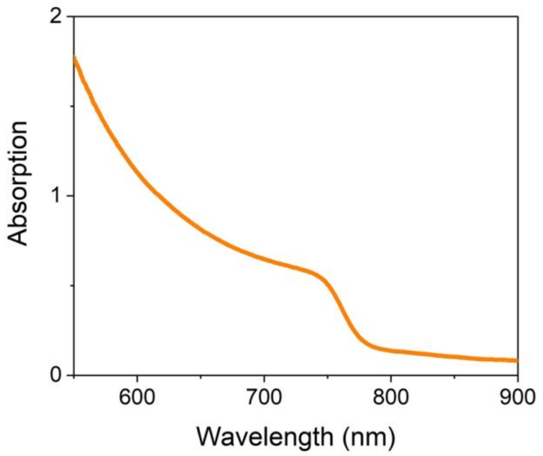

Figure S2: Absorption spectrum of $MAPbI_3$ from a methylamine in acetonitrile precursor deposited on quartz

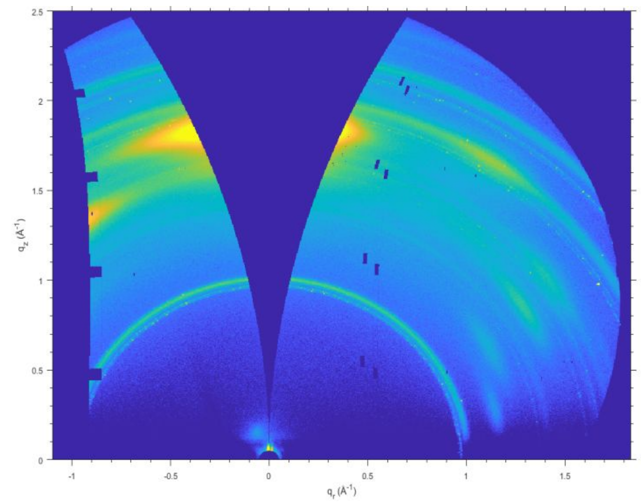

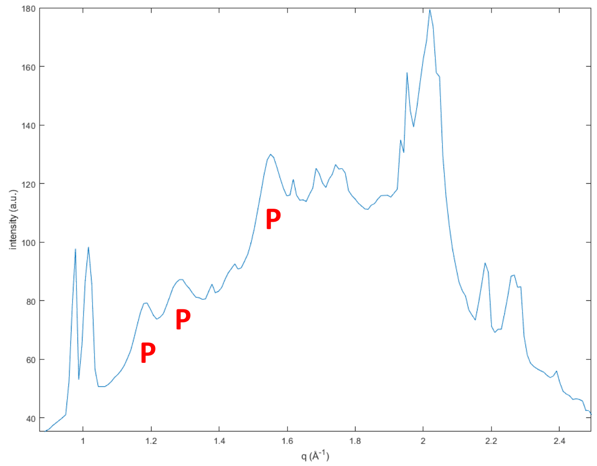

Figure S3: GIWAXS and 1D integrated intensity plot of $MAPbI_3$ in grooves, taken with X-ray path parallel to the direction of the grooves. Note the characteristic lead iodide and perovskite peaks around q=1. Asymmetric scattering pattern caused by plastic substrate (labelled “P”)

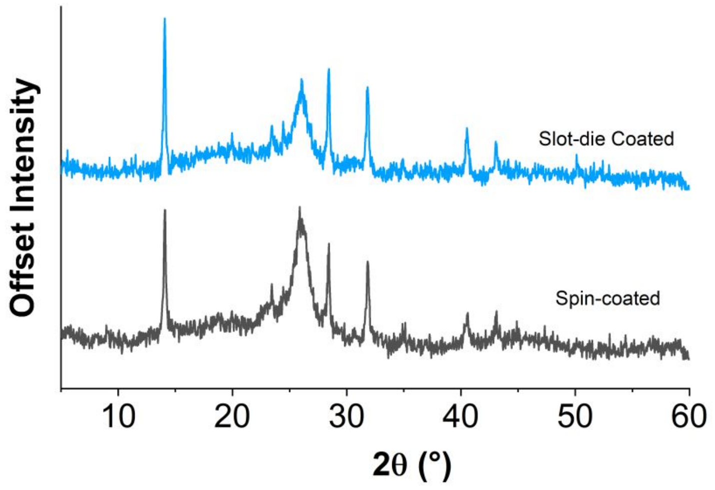

Figure S4: XRD spectra of grooves with the perovskite either slot-die coated and spin coated.

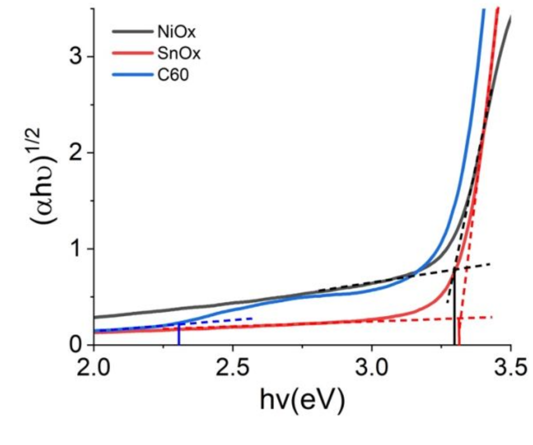

Figure S5: Tauc plots calculated from UV-visible spectra and used to find band gaps in C60, SnOx and NiOx

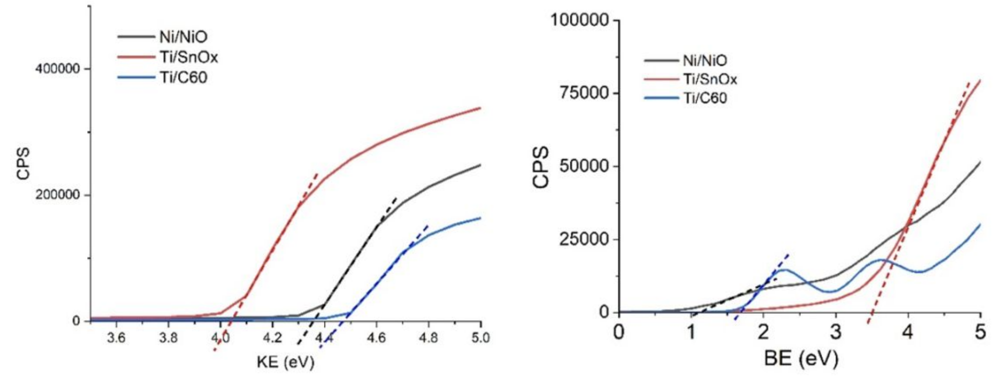

Figure S6: UPS spectra used to find valence band relative to Fermi level in Ni/NiO, SnOx and C_{60}.

Sample	Calculated				Measured		
	Work Function (Eseco KE, eV)	Evb vs Ef (UPS, BE eV)	Evb vs Ef (XPS, BE eV)	Band Gap (UV-Vis, eV)	Evb vs Evac (UPS only eV)	Work Function (XPS & UPS eV)	Ecb vs Evb (eV)
**Ni/NiOx**	4.1	1.1	0.8	3.3	5.2	4.4	1.9
**Ti/SnOx/C60**	4.4	1.9	1.4	2.25	6.3	4.9	4.05
**Ti/SnOx**	3.9	3.6	0.4	3.3	7.5	7.1	4.2

Figure S7: Table of calculated and measured band positions used to construct the band diagram shown in Fig 1.

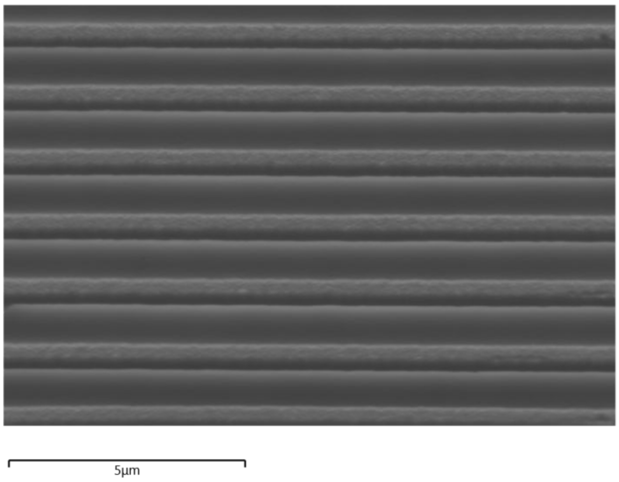

Figure S8: SEM image of a series of grooves that have had the various n and p-type contact layers deposit onto opposing walls. This image was recorded before the deposition of the perovskite.

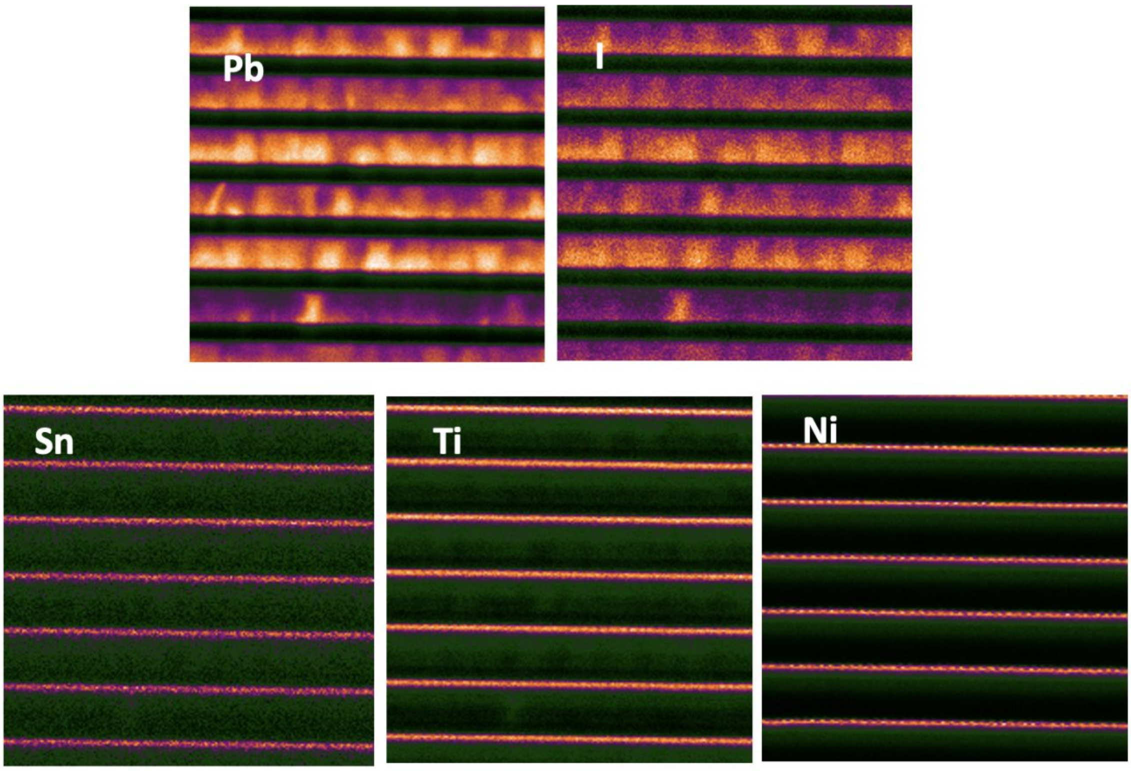

Figure S9: X-ray Fluorescence Mapping of each individual element for grooves with slot die coated perovskite.

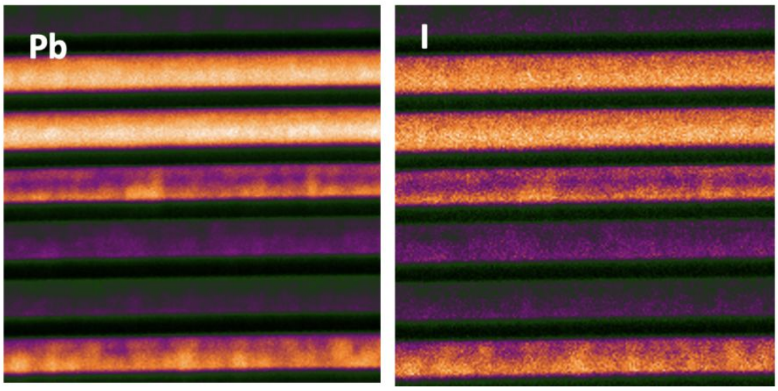

Figure S10: X-ray fluorescence maps of individual elements for grooves with spin-coated perovskite

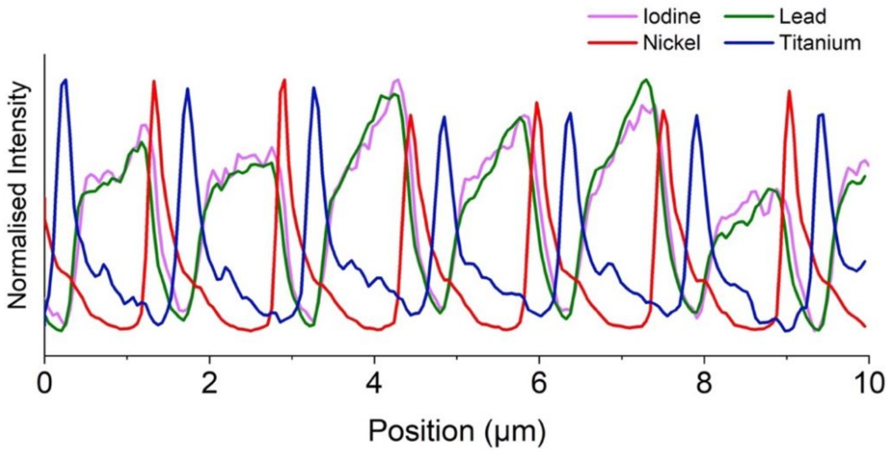

Figure S11: Normalised intensity of each element integrated right to left across the XRF map for the slot-die coated groove devices, taken from data in Fig 3(c).

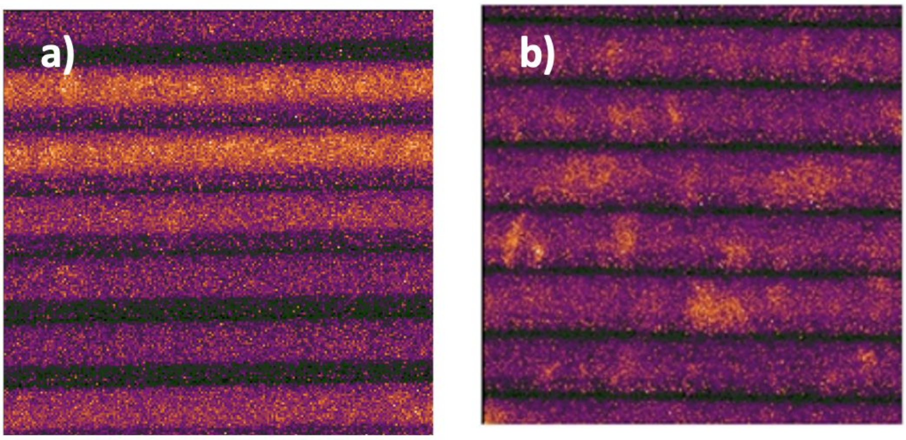

Figure S12: Pb:I ratio of grooves with perovskite either; a) spin-coated or b) slot-die coated.

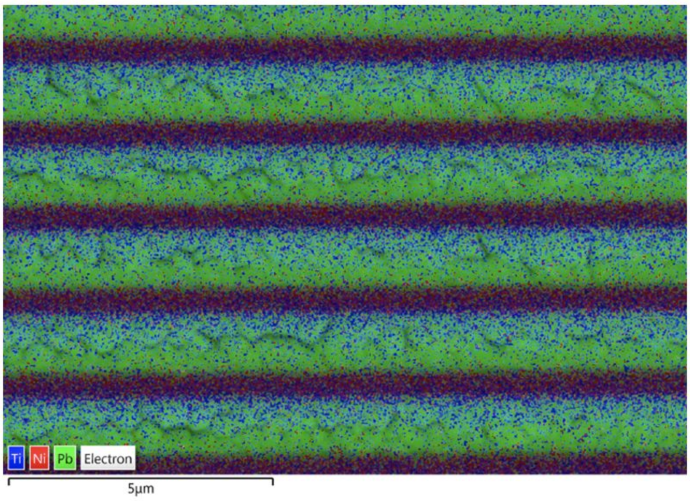

Figure S13: EDX SEM maps of each element in perovskite filled grooves, with perovskite deposited via slot die-coating. Image overlayed with CBS image.

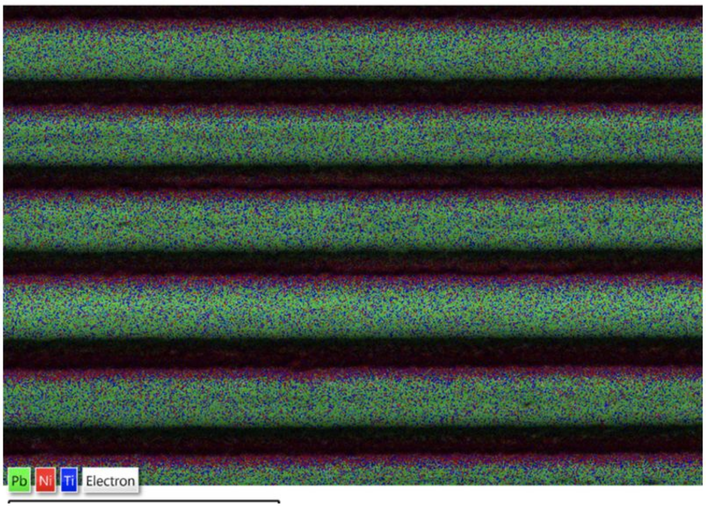

Figure S14: EDX SEM maps of each element in perovskite filled grooves, with perovskite deposited via spin-coating. Image overlayed with CBS image.

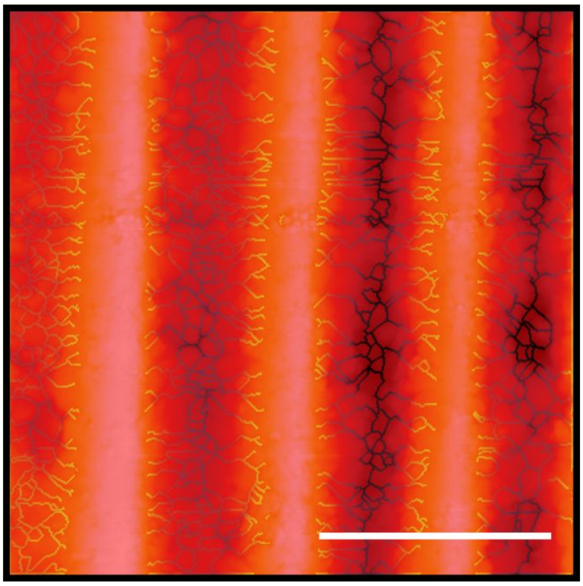

Figure S15: Image showing mask used to separate grains in AFM image in 3d. Flat sections were first masked off, then grain boundaries segmented by watershed. Scale bar 2 µm

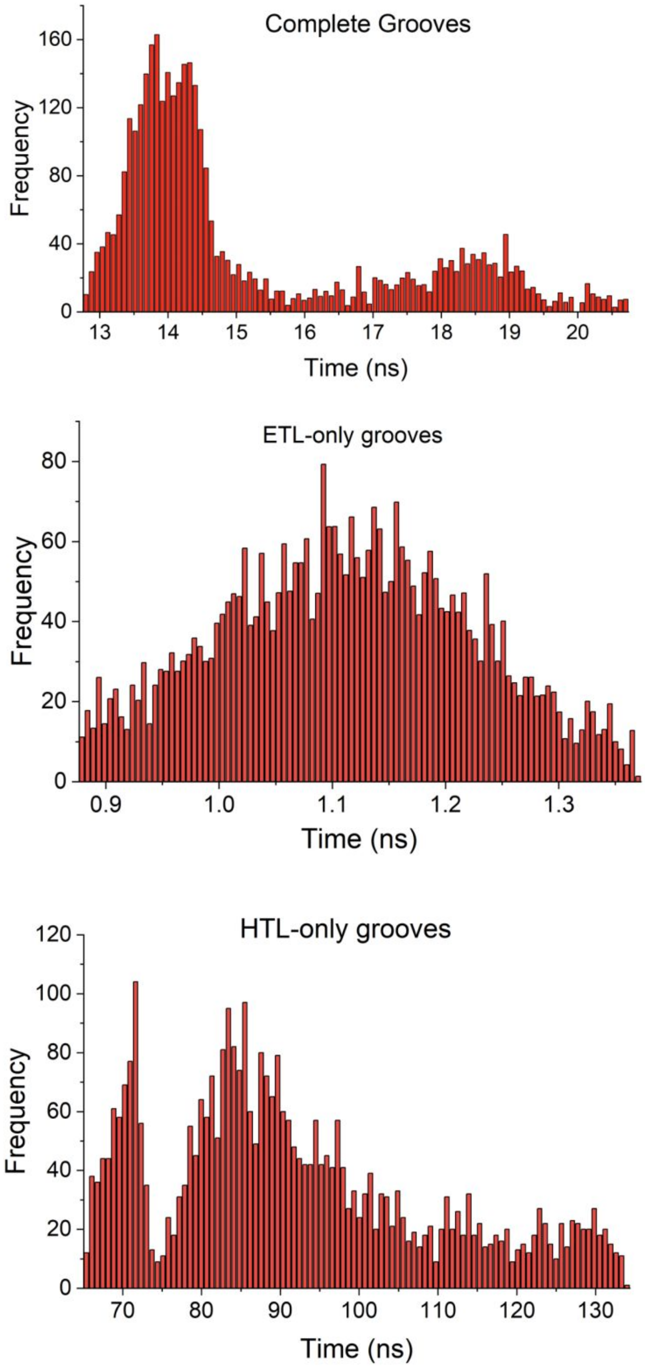

Figure S16: Histogram of a series of fluorescence lifetimes taken recorded for a $MAPbI_3$ perovskite on groove substrates. Here, either one or both groove walls were coated using the different transport materials (HTL: NiO/Ni, ETL: $C_{60}/SnO_x/Ti$). The $MAPbI_3$ perovskite was deposited by slot die coating.

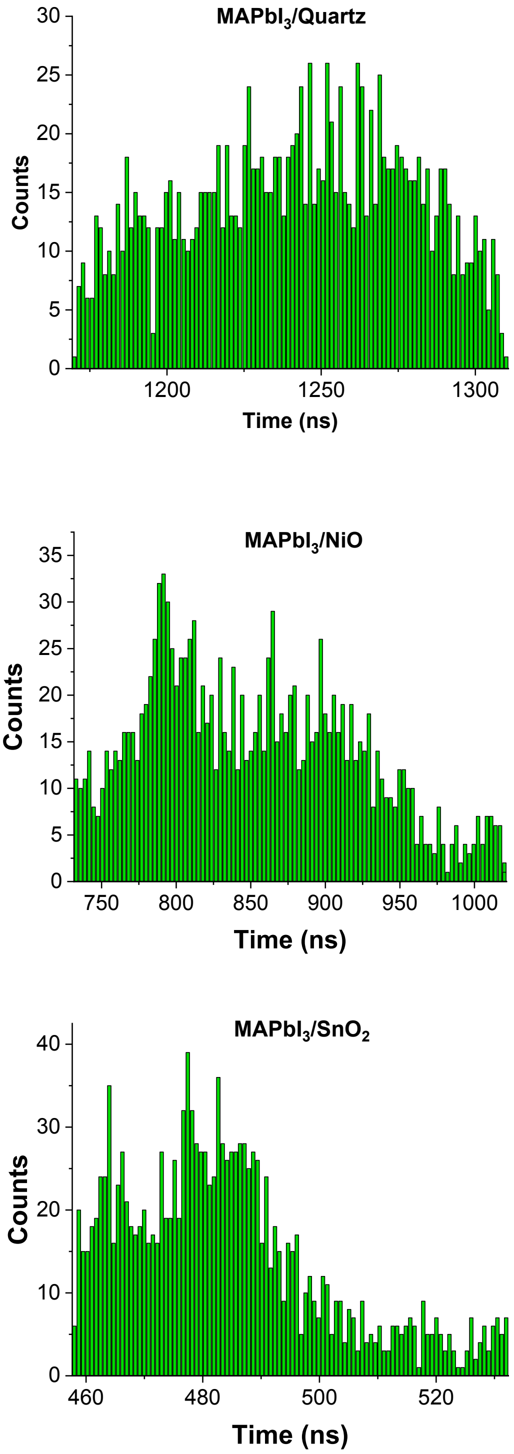

Figure S17: Histogram of a series of fluorescence lifetimes of a $MAPbI_3$ perovskite on a quartz substrate, and quartz-substrates coated with the different electron and hole extraction materials. Here, the $MAPbI_3$ perovskite was deposited by spin coating.

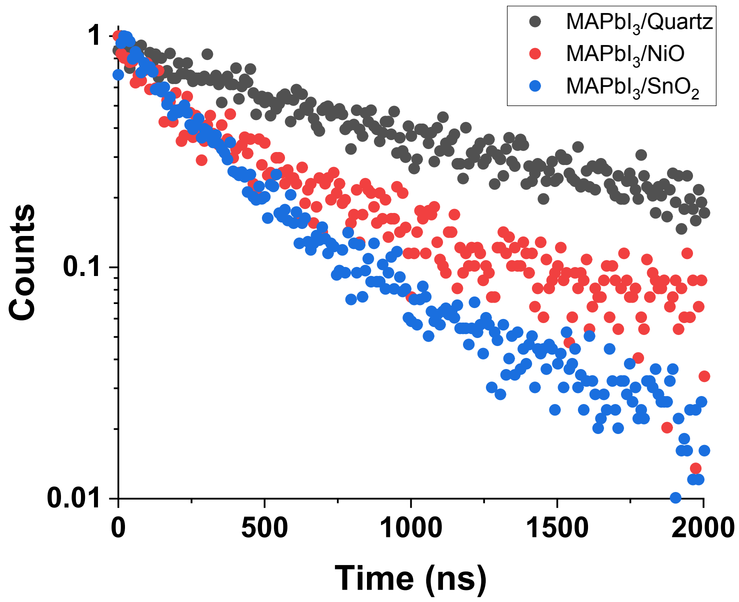

Figure S18: Example emission decay curves for $MAPbI_3$ films deposited by spin coating onto a quartz- substrate, or quartz-substrates coated with SnO_x or NiO.

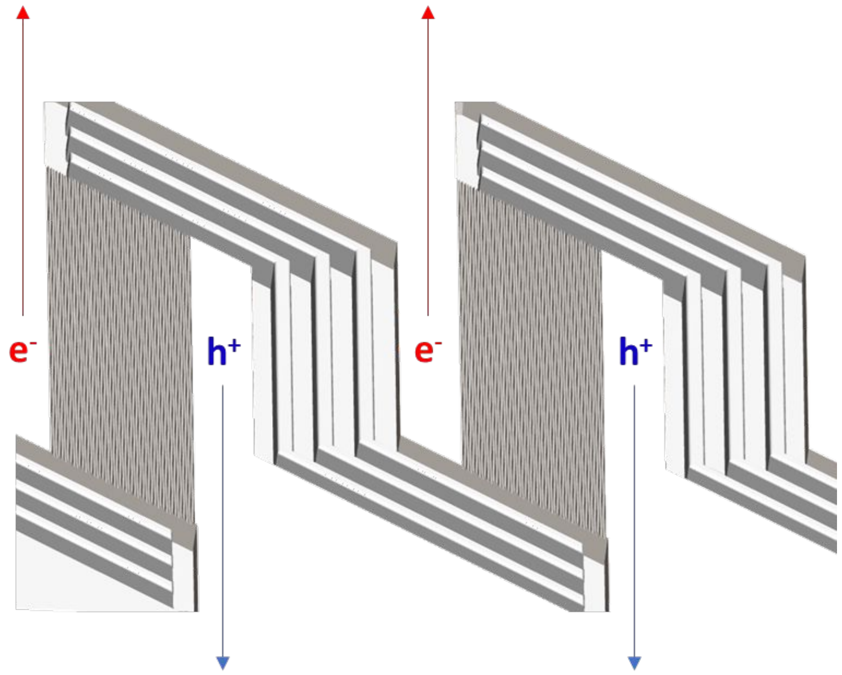

Figure S19: Layout of cascades and resistive delineation features that separate cascades and allow their parallel connection when bus bars are attached across the top and bottom.

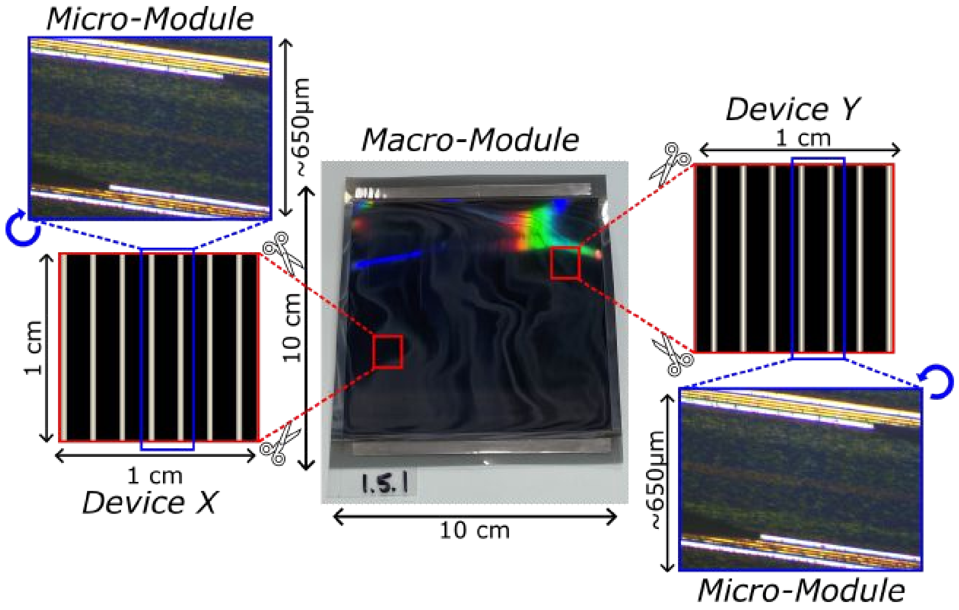

Figure S20: Schematic showing selection of devices for study from a macro-module.

	**n-*i*-p**	**p-*i*-n**
**PCE (%)**	16.3	11.3
**Jsc (mA/cm²)**	21.17	17.14
**Voc (V)**	1.09	1.01
**FF (%)**	70.4	65.8

Figure S21: Champion reverse sweep performance metrics of closest equivalent flat devices to groove structure. Here, n-*i*-p devices correspond to the structure ITO/SnO_2/$MAPbI_3$/Spiro-OMeTAD/Au. p-*i*-n devices correspond to the architecture ITO/NiO/$MAPbI_3$/C_{60}/BCP/Ag. The $MAPbI_3$ perovskite precursor was based on the formulation first introduced by Noel et al [7].

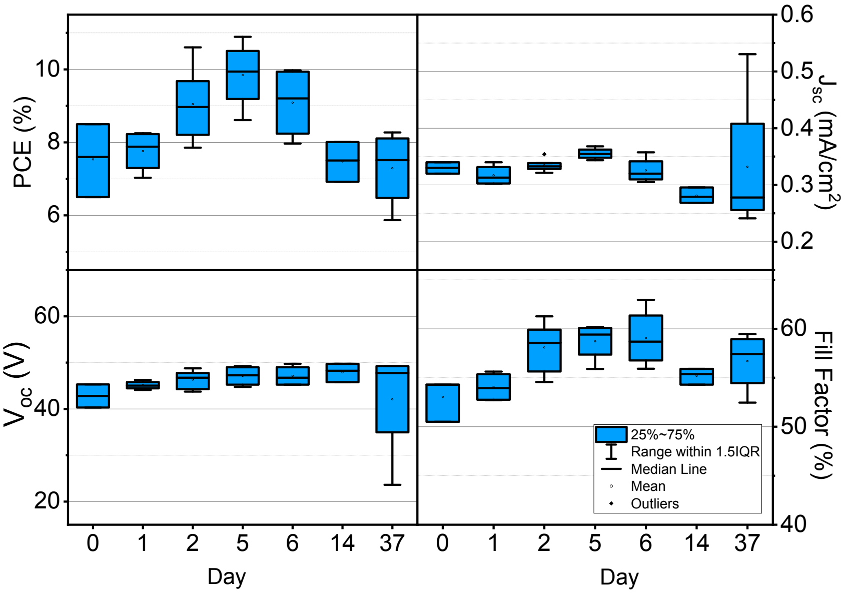

Figure S22: Box plots of performance metrics for a set of 50 groove devices measured periodically over the course of 37 days. Note, these devices were not fabricated on a moving web, but the transport layers were coated at a glancing angle and were in fixed in place relative to the deposition source. The perovskite was then deposited via spin-coating.
